# Preliminary prediction of semen quality based on modifiable lifestyle factors by using the XGBoost algorithm

**DOI:** 10.3389/fmed.2022.811890

**Published:** 2022-09-13

**Authors:** Mingjuan Zhou, Tianci Yao, Jian Li, Hui Hui, Weimin Fan, Yunfeng Guan, Aijun Zhang, Bufang Xu

**Affiliations:** ^1^Department of Obstetrics and Gynecology, Ruijin Hospital, Shanghai Jiao Tong University School of Medicine, Shanghai, China; ^2^Shanghai National Engineering Research Center of Digital Television Co., Ltd., Shanghai, China; ^3^Clinical Research Center, Ruijin Hospital, Shanghai Jiao Tong University School of Medicine, Shanghai, China; ^4^Cooperative Medianet Innovation Center, Shanghai Jiao Tong University, Shanghai, China; ^5^School of Electronic Information and Electrical Engineering, Shanghai Jiao Tong University, Shanghai, China; ^6^Department of Histo-Embryology, Genetics and Developmental Biology, Shanghai Key Laboratory of Reproductive Medicine, Shanghai Jiao Tong University School of Medicine, Shanghai, China

**Keywords:** lifestyles, semen quality, artificial intelligence, machine learning, extreme gradient boosting (XGBoost)

## Abstract

**Introduction:**

Semen quality has decreased gradually in recent years, and lifestyle changes are among the primary causes for this issue. Thus far, the specific lifestyle factors affecting semen quality remain to be elucidated.

**Materials and methods:**

In this study, data on the following factors were collected from 5,109 men examined at our reproductive medicine center: 10 lifestyle factors that potentially affect semen quality (smoking status, alcohol consumption, staying up late, sleeplessness, consumption of pungent food, intensity of sports activity, sedentary lifestyle, working in hot conditions, sauna use in the last 3 months, and exposure to radioactivity); general factors including age, abstinence period, and season of semen examination; and comprehensive semen parameters [semen volume, sperm concentration, progressive and total sperm motility, sperm morphology, and DNA fragmentation index (DFI)]. Then, machine learning with the XGBoost algorithm was applied to establish a primary prediction model by using the collected data. Furthermore, the accuracy of the model was verified *via* multiple logistic regression following *k*-fold cross-validation analyses.

**Results:**

The results indicated that for semen volume, sperm concentration, progressive and total sperm motility, and DFI, the area under the curve (AUC) values ranged from 0.648 to 0.697, while the AUC for sperm morphology was only 0.506. Among the 13 factors, smoking status was the major factor affecting semen volume, sperm concentration, and progressive and total sperm motility. Age was the most important factor affecting DFI. Logistic combined with cross-validation analysis revealed similar results. Furthermore, it showed that heavy smoking (>20 cigarettes/day) had an overall negative effect on semen volume and sperm concentration and progressive and total sperm motility (OR = 4.69, 6.97, 11.16, and 10.35, respectively), while age of >35 years was associated with increased DFI (OR = 5.47).

**Conclusion:**

The preliminary lifestyle-based model developed for semen quality prediction by using the XGBoost algorithm showed potential for clinical application and further optimization with larger training datasets.

## Introduction

Semen quality is an important determinant of male fertility (1, 2). In recent years, the semen quality has decreased, and this adverse trend has aroused widespread concern (3, 4). Many factors have been reported to affect semen quality, including demographic characteristics such as age and body mass; diseases such as endocrine or genetic problems, prostate disorders, seminal tract obstruction, and oncological diseases; environmental factors such as temperature changes, pollution, and electromagnetic radiation; and lifestyle factors such as smoking, alcohol intake, and staying up late (1, 5–10). Extensive research has indicated that unhealthy lifestyles are among the most important factors accounting for male reproductive disorders and decreased semen quality (6, 11). However, the specific lifestyle factors affecting semen quality remain to be elucidated. Furthermore, undertaking the relevant research required for this purpose is difficult because of lifestyle complexity (characterized by factors such as frequent changes or the involvement of various characteristics and confounding variables).

Machine learning, a branch of artificial intelligence (AI), is suitable for dealing with flexible relationships among predictor variables and outcomes in large datasets (12). The application of machine learning in multiple fields of medicine could help develop disease prediction models (13–15), and many studies have applied this approach to the analysis of semen parameters, such as morphology (16). However, there are few studies involving the application of AI in the prediction of the impact of lifestyles on semen quality. To our knowledge, thus far, only 2 small-sample (*n* = 100) studies have revealed the effects of lifestyle variations on semen parameters (17, 18). Furthermore, the volunteers recruited in these studies were young (age between 18 and 36 years) and the semen quality parameters included were limited or ambiguous. Therefore, further comprehensive and extensive research is warranted. Hence, in this study, XGBoost, a decision-tree based machine learning algorithm, was applied to analyze the association between the semen quality characteristics and the lifestyles associated by using data collected from 5,109 men examined in our reproductive medical center, so as to develop a preliminary model for semen quality prediction. Furthermore, the accuracy of the model was verified *via* multiple logistic regression analyses to determine the value of further study.

## Materials and methods

### Study design

**Figure 1:**
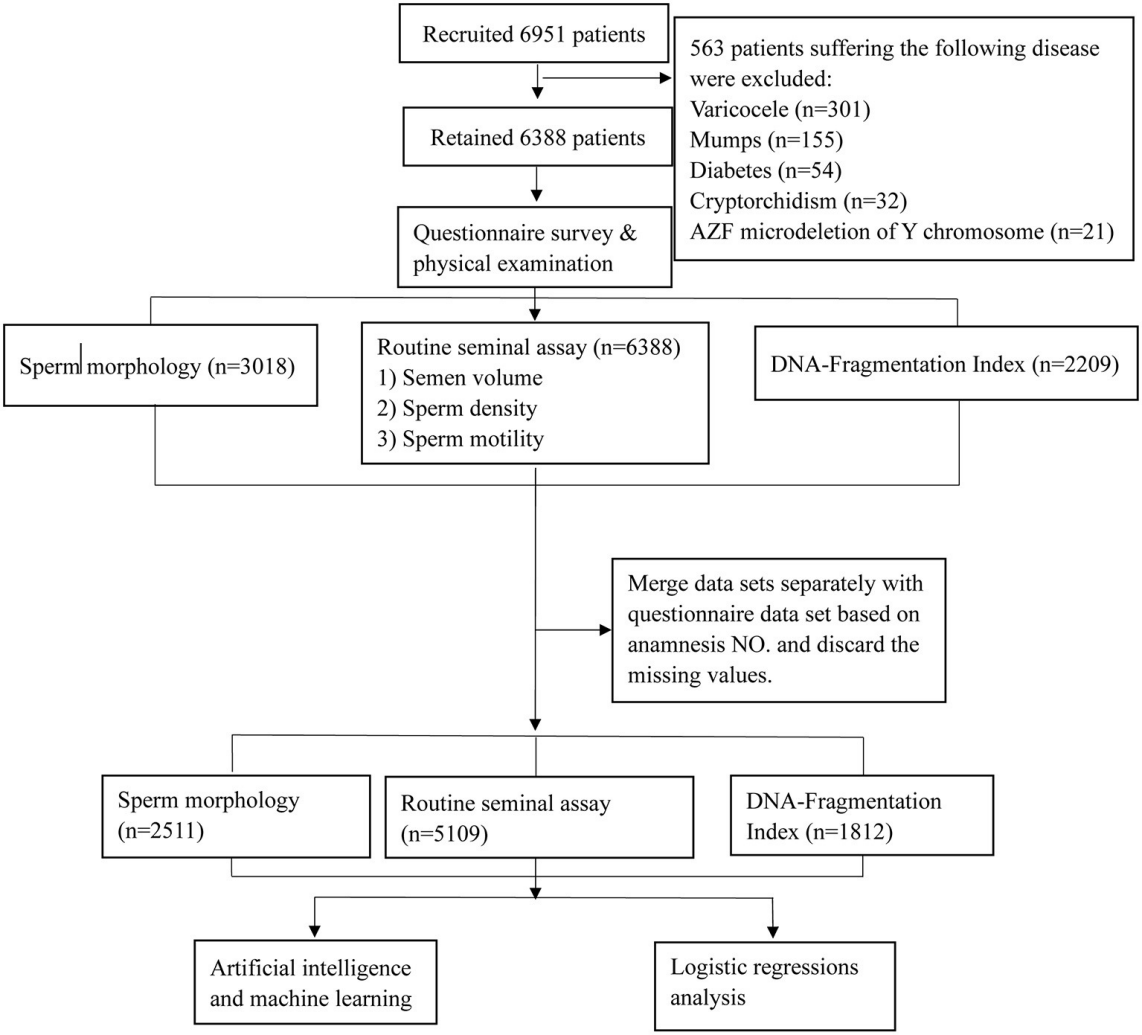
Flow chart of the study population. A total of 5,109 males were included in this study; all of these participants underwent a routine seminal assay while some also underwent sperm morphology and DNA Fragmentation Index assay according to the different inspection purposes.

This study was approved by the Ethics Committee of Ruijin Hospital, School of Medicine, Shanghai Jiao Tong University (No. 2019-185), and all participants recruited signed informed consent forms. As shown in the patient recruitment flowchart (Figure 1), from October 2019 to September 2021, 6,951 men examined in our center were recruited. Participants with a BMI < 32 and without chromosome abnormalities were included. The exclusion criteria were as follows: prostatic inflammation and organic injury, seminal tract obstruction, cancer, hypospadias, low testosterone levels, varicocele, mumps, cryptorchidism, diabetes, microdeletion of the Y chromosome azoospermia factor (AZF), hyperlipidemia, hypertension, and sexually transmitted diseases. Ultimately, 6,388 men were included after dropping 563 patients who met the exclusion criteria. According to the different evaluations intended, the routine seminal assay including semen volume, sperm concentration, and sperm motility, was performed for all participants, while sperm morphology tests and DNA fragmentation index (DFI) examination was performed in 3,018 and 2,209 participants, respectively. Each participant completed a baseline questionnaire before the semen analysis, and cases with missing questionnaire responses were excluded. Thus, the final dataset included 5,109 men whose semen volume, sperm concentration, and sperm motility were analyzed. Furthermore, sperm morphology and DFI analyses were performed in 2,511 and 1,812 participants, respectively.

### Questionnaire variables

The questionnaire comprised 13 items including 10 pertaining to habitual lifestyles and three general conditions including age, abstinence period, and date of questionnaire completion; the details are listed in Supplementary Table 1. Sleeplessness and intensity of sports activity were analyzed using the Insomnia Severity Index (Supplementary Table 2) and modified Physical Activity Questionnaire (Supplementary Table 3) (19), respectively.

### Assessment of semen quality

Semen samples were collected in sterile plastic container by asking the participants to masturbate. The participants were asked to void urine and wash their hands and external genitalia before masturbating to provide the sample. The sample collected was placed in a water bath maintained at 37°C for 30–60 min for liquefaction. Semen volume was measured by weighing, assuming a semen density of 1.0 g/ml. Sperm concentration (spermatozoa N/mL) and motility (%) were evaluated using a computer-aided sperm analysis system. DFI was determined by flow cytometry after staining with acridine orange, and sperm morphology was investigated using the Diff-Quick staining method. Reference values from the World Health Organization semen analysis manual were used to assess semen characteristics (20), and values below the lower threshold provided in the WHO manual were defined as abnormal. Besides, the threshold of DFI 30% was applied to classify normal (DFI < 30%) or abnormal (DFI ≥ 30%) groups according to a previously published article (21).

### AI and machine learning

The algorithm used in this study was extreme gradient boosting (XGBoost). The feature importance was calculated by the gain method from the XGBoost python library, which worked by averaging training loss reduction caused by feature utilization for each splitting. The input variables were the information collected from the questionnaire of each patient, and the output variables were the semen quality parameters. The input variables were considered categorical variables (Supplementary Table 1), and the output variables were considered dichotomous variables according to the criterion described above. The six semen quality parameters were independent indicators; the XGBoost model was developed using different hyperparameters, separately, to improve the accuracy of the algorithm.

Cross-validation was performed to adjust the parameters. First, a relatively high “learning_rate” was used and the optimum “n_estimators” was selected for this “learning_rate”. Secondly, the parameters “max_depth” and “min_child_weight” were adjusted for the selected “learning_rate” and “n_estimators.” Owing to the unbalanced category of the dataset, the training dataset was oversampled, and the “scale_pos_weight” was always equal to 1. Then, the learning rate was reduced. Next, “max_depth” was adjusted to simplify the XGBoost model according to the results obtained for the test dataset. The clean dataset used for XGBoost was randomly split into training and test datasets in a ratio of 70:30. Hyperparameter details are described in Table 1. Lastly, *k*-fold cross-validation with *k* = 10 was performed to evaluate machine learning models.

**Table 1:** Distribution of male participants whose data were used for machine learning and the hyperparameters for XGBoost.

**Sperm quality parameters**	**Total no**.	**Train set no**.	**Test set no**.	**Learning rate**	***N* estimators**	**Max depth**	**Min_child_weight**	**Scale_pos_weight**
Semen volume	5,109	3,576	1,533	0.01	600	3	1	1
Sperm concentration	5,109	3,576	1,533	0.01	750	3	1	1
Progressive motility	5,109	3,576	1,533	0.01	600	3	1	1
Total motility	5,109	3,576	1,533	0.01	600	5	1	1
Sperm morphology	2,511	1,758	754	0.01	300	4	1	1
DFI	1,812	1,268	544	0.01	300	4	1	1

### Statistical analysis and logistic regression analysis

Descriptive statistics were used to summarize general demographics. The correlations among 13 questionnaire items were evaluated by Pearson's correlation coefficients. For continuous variables, data are expressed as mean ± SD for normally distributed data or median (Interquartile range, IQR) values for non-parametric data. For categorical variables, data are expressed as percentages.

Univariate and multivariable logistic regression was used to identify the factors related to semen quality. For each independent variable, odds ratios (ORs) and 95% confidence intervals (CIs) were estimated. Collinearity analyses were performed before the logistic regression analysis, and the model's goodness-of-fit was graphically evaluated (ROC curves). The response variables were categorized per the method used for the XGBoost algorithm, and stepwise regression was applied for all multivariate logistic regression analyses. Moreover, *k*-fold cross-validation with *k* = 10 was performed to evaluate the accuracy of the model.

The univariate and multivariable logistic regression analyses were performed with SAS version 9.4 (SAS Institute, Cary, NC, USA), and k-fold cross-validation was performed with *k* = 10 by using the package for R (version 4.1.2). Other statistical analyses were performed using SPSS 23.0. *P* < 0.05 was considered statistically significant.

## Results

### General information collected using the questionnaire

**Figure 2:**
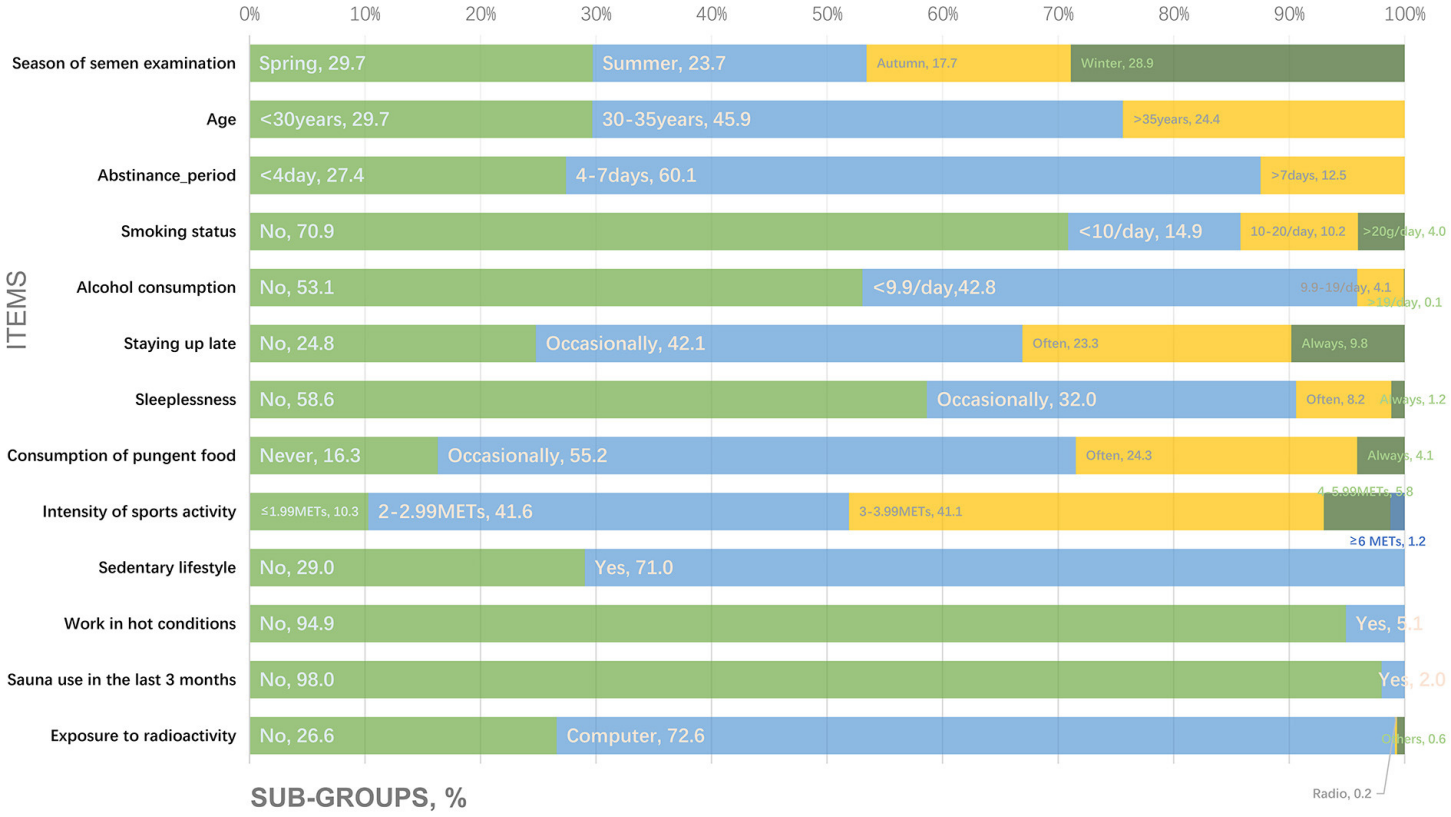
Information regarding the general and lifestyle characteristics of study participants.

Figure 2 shows the proportions of participants corresponding to the subgroups for the following questionnaire items: (1) season of semen examination; (2) age; (3) abstinence period; (4) smoking status; (5) alcohol consumption; (6) staying up late; (7) sleeplessness; (8) consumption of pungent food; (9) intensity of sports activity; (10) sedentary lifestyle; (11) work in hot conditions; (12) sauna use in the last 3 months; and (13) exposure to radioactivity.

### Semen quality among study participants

Among the 5,109 males, the median semen volume, sperm concentration, total sperm count, rapid progressive motility of the sperm, progressive motility of the sperm, and total sperm motility were 3.3 ml (95% CI: 3.40–3.49 ml), 68.1 × 10^6^/ml (95% CI: 79.26–82.84 × 10^6^/ml), 214.5 × 10^6^ (95% CI: 262.28–275.23 × 10^6^), 23.0% (95% CI: 22.76–23.50%), 47.8% (95% CI: 45.50–46.74%), and 60.4% (95% CI: 56.46–57.85%), respectively. The median normal sperm morphology among 2,511 men was 6.0% (95% CI: 6.24–6.51%), and the median DFI of 1,915 men was 14.4% (95% CI: 17.29–18.39%). In addition, 18.2% of the participants showed abnormal sperm morphology (morphologically normal forms, <4.0%, *n* = 2,511) and 13.9% had high DFI (≥30%, *n* = 1,812).

### Risk factors affecting semen volume

We trained XGBoost with the input of the 13 items and achieved 60.7–70.3% accuracy, 55.4–72.5% sensitivity, and 39.9–70.4% specificity for the test set (Table 2).

**Table 2:** Outcomes of machine learning using XGboost.

**Sperm quality parameters**	**Classification accuracy**	**True negative**	**False positive**	**False negative**	**True positive**	**Sensitivity**	**Specificity**	**Positive predictive value**	**Negative predictive value**
Semen volume	0.7025	63	71	385	1,014	0.7248	0.4701	0.9346	0.1406
Sperm concentration	0.6758	94	87	410	942	0.6967	0.5193	0.9155	0.1865
Progressive motility	0.6282	269	147	423	694	0.6213	0.6466	0.8252	0.3887
Total motility	0.6067	218	157	446	712	0.6149	0.5813	0.8193	0.3283
DFI	0.6838	331	139	33	41	0.5541	0.7043	0.2278	0.9093
Sperm morphology	0.6167	55	83	206	410	0.6656	0.3986	0.8316	0.2107

**Figure 3:**
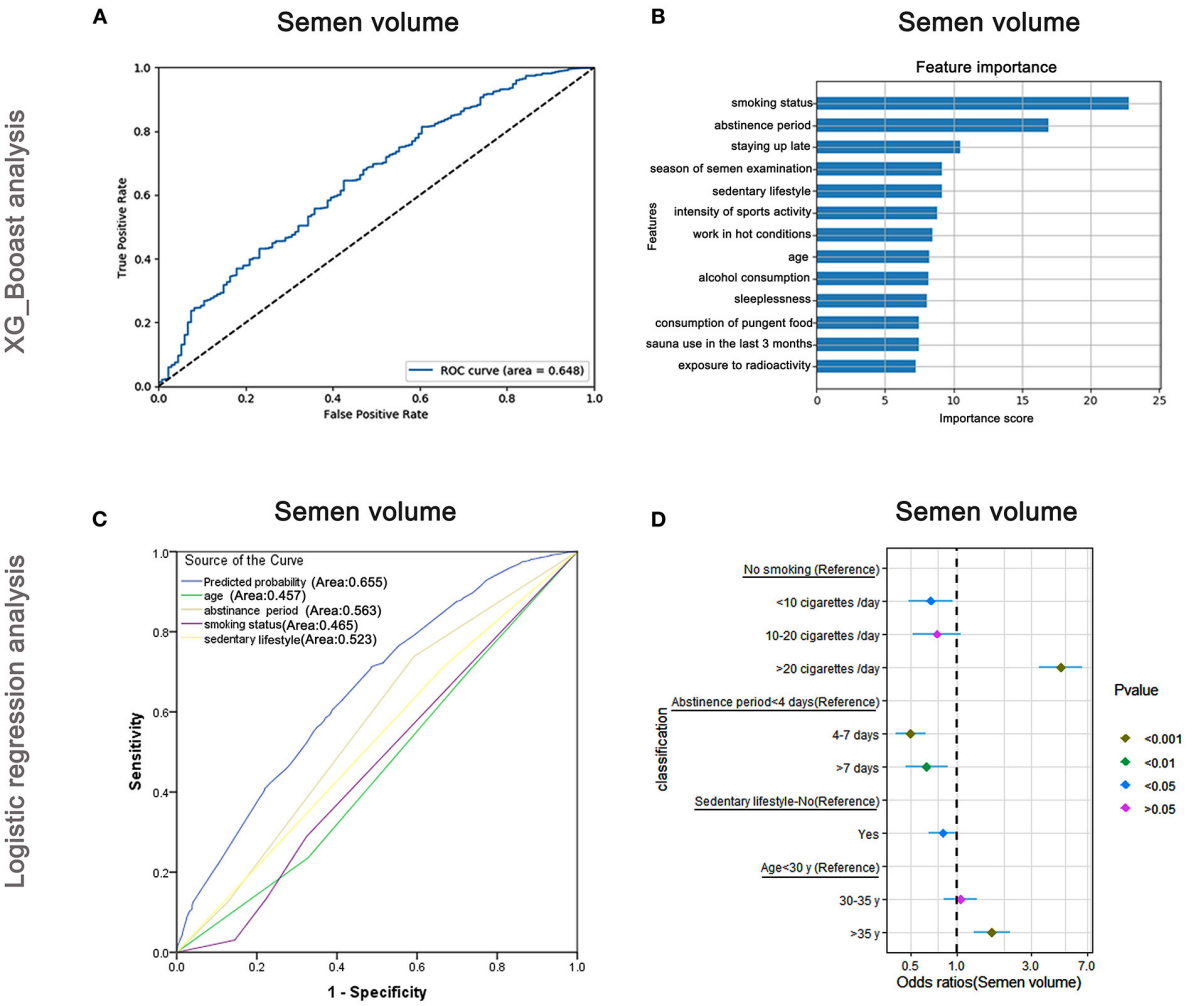
XGBoost and logistic regression analysis of the risk factors for semen volume. The ROC curve **(A)** and feature importance **(B)** analyzed by XGBoost and the ROC curve **(C)** and forest diagram showing significant risk factors **(D)** analyzed by logistic regression.

The AUC of the XGBoost model for semen volume was 0.648 (Figure 3A) and the following cross-validation showed that the AUC of the model was 0.617. The feature importance plotted *via* XGBoost showed that the maximum score was for smoking status followed by abstinence period and staying up late (Figure 3B). Logistic regression analyses (Figure 3C) revealed that smoking status, abstinence period, sedentary lifestyle, and age were predictive markers of semen volume. The AUC of the combined markers (AUC = 0.655) was higher than that of the individual markers (AUC = 0.465, 0.563, 0.523, and 0.457, respectively), and the following cross-validation based on the multivariate regression analysis showed that the AUC of the model was 0.539. The maximum odds ratio was related to smoking status (OR = 4.69), indicating it to be the most important predictor (Supplementary Table 4). Abstinence period, the second most important factor as revealed by XGBoost, was significantly associated with semen volume in the logistic regression analysis (Supplementary Table 4). Besides, as shown in Figure 3D, the OR per the regression analysis indicated that men who smoked more than 20 cigarettes/day were more likely to have a lower semen volume (OR: 4.69, 95% CI: 3.39–6.49, *P* < 0.001). However, males who smoked <10 cigarettes/day were less likely to have a lower semen volume (OR: 0.67, 95% CI: 0.48–0.93, *P* < 0.05) than non-smokers. Men who practiced abstinence for more than 7 days or had a sedentary lifestyle (≥5 h/day) were less likely to have a lower semen volume (OR: 0.63, 95% CI: 0.46–0.87, *P* < 0.01 and OR: 0.81, 95% CI: 0.65–1.00, *P* < 0.05, respectively).

### Risk factors affecting sperm concentration

**Figure 4:**
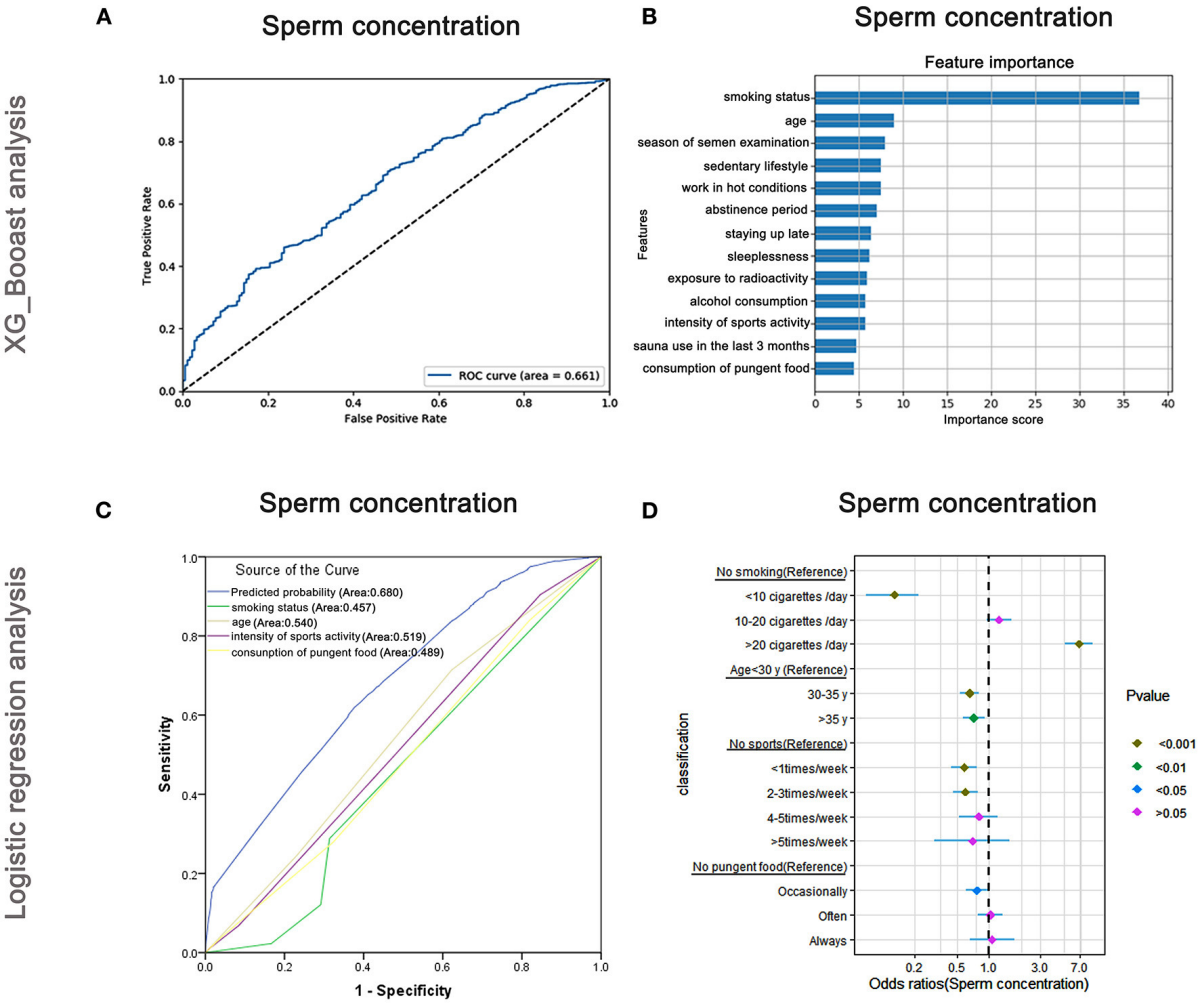
XGBoost and logistic regression analysis of the risk factors for sperm concentration. The ROC curve **(A)** and feature importance **(B)** analyzed by XGBoost and the ROC curve **(C)** and forest diagram showing significant risk factors **(D)** analyzed by logistic regression.

The AUC of the XGBoost model for sperm concentration was 0.661 (Figure 4A), and the cross-validation showed that the AUC of the model was 0.674. The feature importance plotted using XGBoost showed that the maximum important score was for smoking status, followed by age and season of semen examination (Figure 4B). The AUCs of the logistic regression analyses (Figure 4C) revealed that smoking status, age, intensity of sports activity, and consumption of pungent food were predictive markers of sperm concentration. The AUC of the combined marker (AUC = 0.680) was higher than those of individual markers (AUC = 0.457, 0.540, 0.519, and 0.489, respectively), and the cross-validation based on the multivariate regression analysis showed that the AUC of the model was 0.547. The maximum odds ratio was observed for smoking status (OR = 6.97), indicating it is the most important predictor (Supplementary Table 5). Age, the second-most important factor revealed by XGBoost, also showed significant association with sperm concentration *via* logistic regression assay (Supplementary Table 5). Besides, as shown in Figure 4D, males who smoked more than 20 cigarettes/day were more likely to have lower sperm concentrations than non-smokers (OR: 6.97, 95% CI: 5.18–9.37, *P* < 0.001), but smokers were less likely to have lower sperm concentrations than non-smokers when they smoked < 10 cigarettes/day (OR: 0.13, 95% CI: 0.07–0.22, *P* < 0.001). Older men (>35 years) were less likely to have lower sperm density (OR: 0.72, 95% CI: 0.57–0.91, *P* < 0.01) than younger men (<30 years).

### Risk factors affecting progressive sperm motility

**Figure 5:**
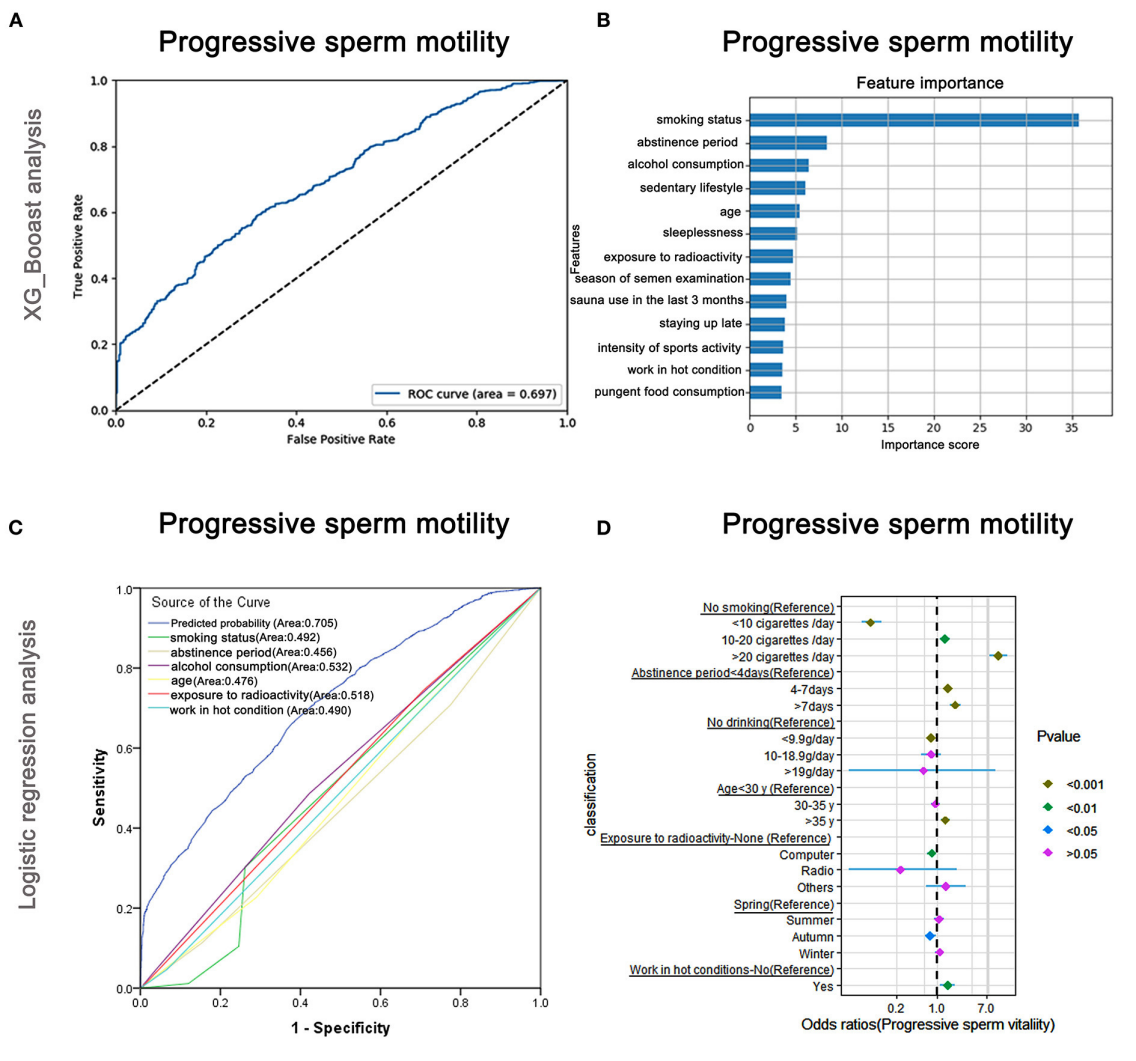
XGBoost and logistic regression analysis of the risk factors for progressive sperm motility. The ROC curve **(A)** and the feature importance **(B)** analyzed by XGBoost and the ROC curves **(C)** and forest diagram showing significant risk factors **(D)** analyzed by logistic regression.

The AUC of the XGBoost models for progressive sperm motility was 0.697 (Figure 5A), and the cross-validation showed that the AUC of the model was 0.698. The feature importance plotted using XGBoost showed that smoking status was the most important factor, followed by abstinence period and alcohol consumption (Figure 5B). The AUCs of the logistic regression analyses (Figure 5C) revealed that smoking status, abstinence period, alcohol consumption, age, exposure to radioactivity, and working in hot conditions were predictive markers of progressive sperm vitality. The AUC of the combined marker (AUC = 0.705) was slightly higher than that of other markers, and the cross-validation based on the multivariate regression analysis showed that the AUC of the model was 0.696, which was similar to that of XGBoost (AUC = 0.697).

The top-two odds ratios were observed for smoking status and abstinence period (OR = 11.16 and 2.05), indicating their importance in predictions (Supplementary Table 6). Alcohol consumption, which was identified as the third-most important by XGBoost, also showed a significant association with progressive sperm motility in the logistic regression assay (Supplementary Table 6).

Moreover, as shown in Figure 5D and Supplementary Table 6, males who smoked more than 20 cigarettes/day were more likely to have lower progressive sperm motility (OR: 11.16, 95% CI: 7.82–15.93, *P* < 0.001) than non-smokers, but smokers were less likely to have lower progressive sperm motility than non-smokers when they smoked <10 cigarettes/day (OR: 0.07, 95% CI: 0.05–0.11, *P* < 0.001). Males who maintained abstinence for more than 7 days were more likely to show lower progressive sperm motility (OR: 2.05, 95% CI: 1.63–2.57, *P* < 0.001).

### Risk factors affecting total sperm motility

**Figure 6:**
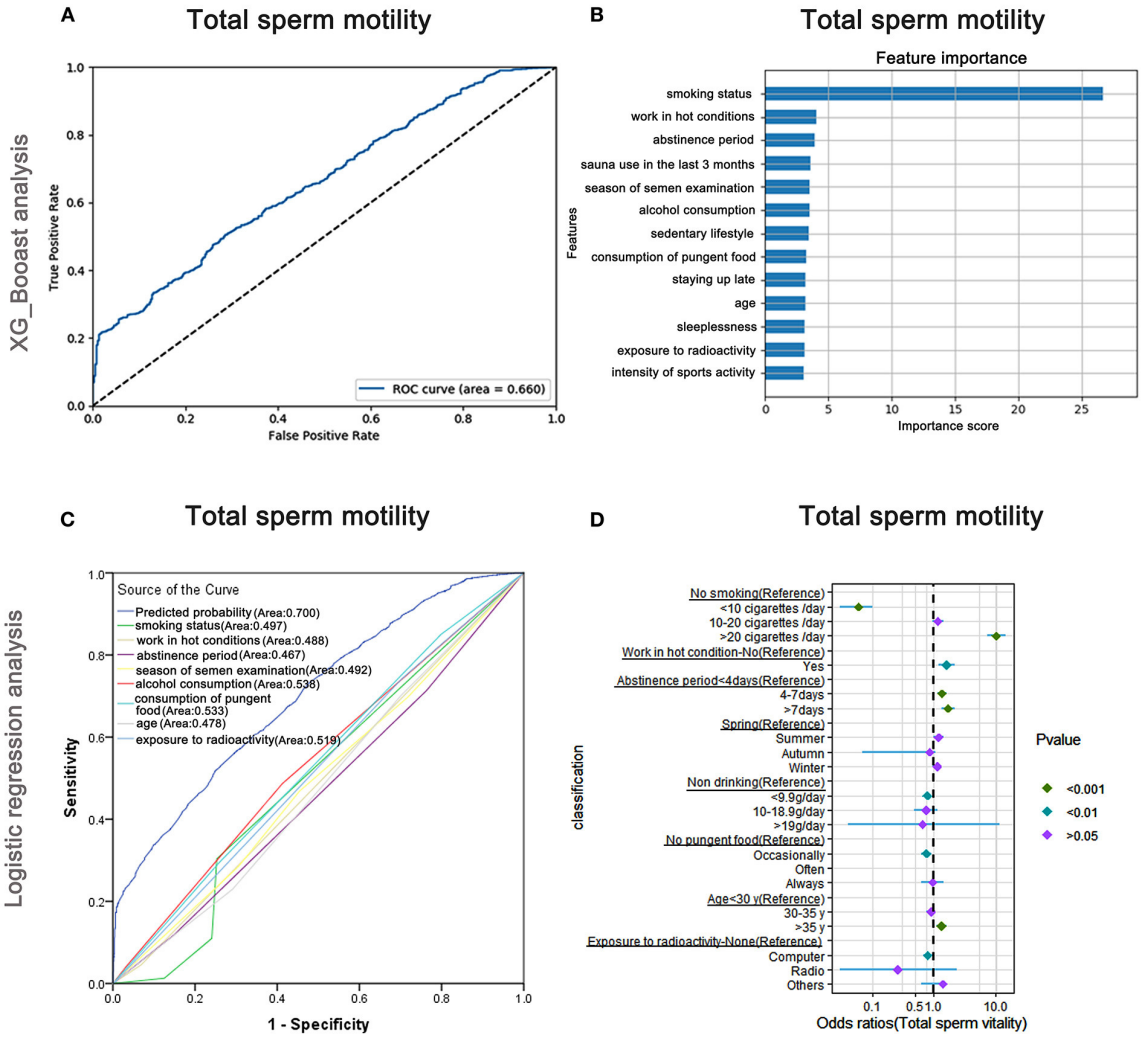
XGBoost and logistic regression analysis of the risk factors for total sperm motility. The ROC curve **(A)** and the feature importance **(B)** analyzed by XGBoost and the ROC curve **(C)** and forest diagram showing significant risk factors **(D)** analyzed by logistic regression.

The AUC of the XGBoost models for total sperm vitalities was 0.660 (Figure 6A), and the cross-validation showed that the AUC of the model was 0.686. The feature importance plotted *via* XGBoost showed that smoking status played the most important part, followed by working in hot conditions and abstinence period (Figure 6B). The AUCs of the logistic regression analyses (Figure 6C) revealed that smoking status, working in hot conditions, abstinence period, season of semen examination, alcohol consumption, consumption of pungent food, age, and exposure to radioactivity were predictive markers of total sperm vitality. The AUC of the combined marker (AUC = 0.700) was slightly higher than that of the other markers, and the cross-validation based on the multivariate regression analysis showed that the AUC of the model was 0.749. The maximum odds ratio was observed for smoking status (OR = 10.35), indicating it was the most important predictor (Supplementary Table 7). Moreover, working in hot conditions and abstinence period, two of the top-three important factors revealed by XGBoost, also significantly affected total sperm motility in the regression analysis (Supplementary Table 7).

As shown in Figure 6D and Supplementary Table 7, males who smoked more than 20 cigarettes/day were more likely to have lower total sperm motility than non-smokers (OR: 10.35, 95% CI: 7.35–14.56, *P* < 0.001), but smokers were less likely to have a lower total sperm motility than non-smokers when they smoked <10 cigarettes/day (OR: 0.06, 95% CI: 0.03–0.10, *P* < 0.001). Males who worked under hot conditions were less likely to show low total sperm motility (OR: 1.63, 95% CI: 1.20–2.21, *P* < 0.05). Moreover, males who maintained abstinence for more than 7 days were more likely to have lower total sperm motility (OR: 1.72, 95% CI: 1.37–2.17, *P* < 0.001).

### Risk factors affecting sperm morphology

**Figure 7:**
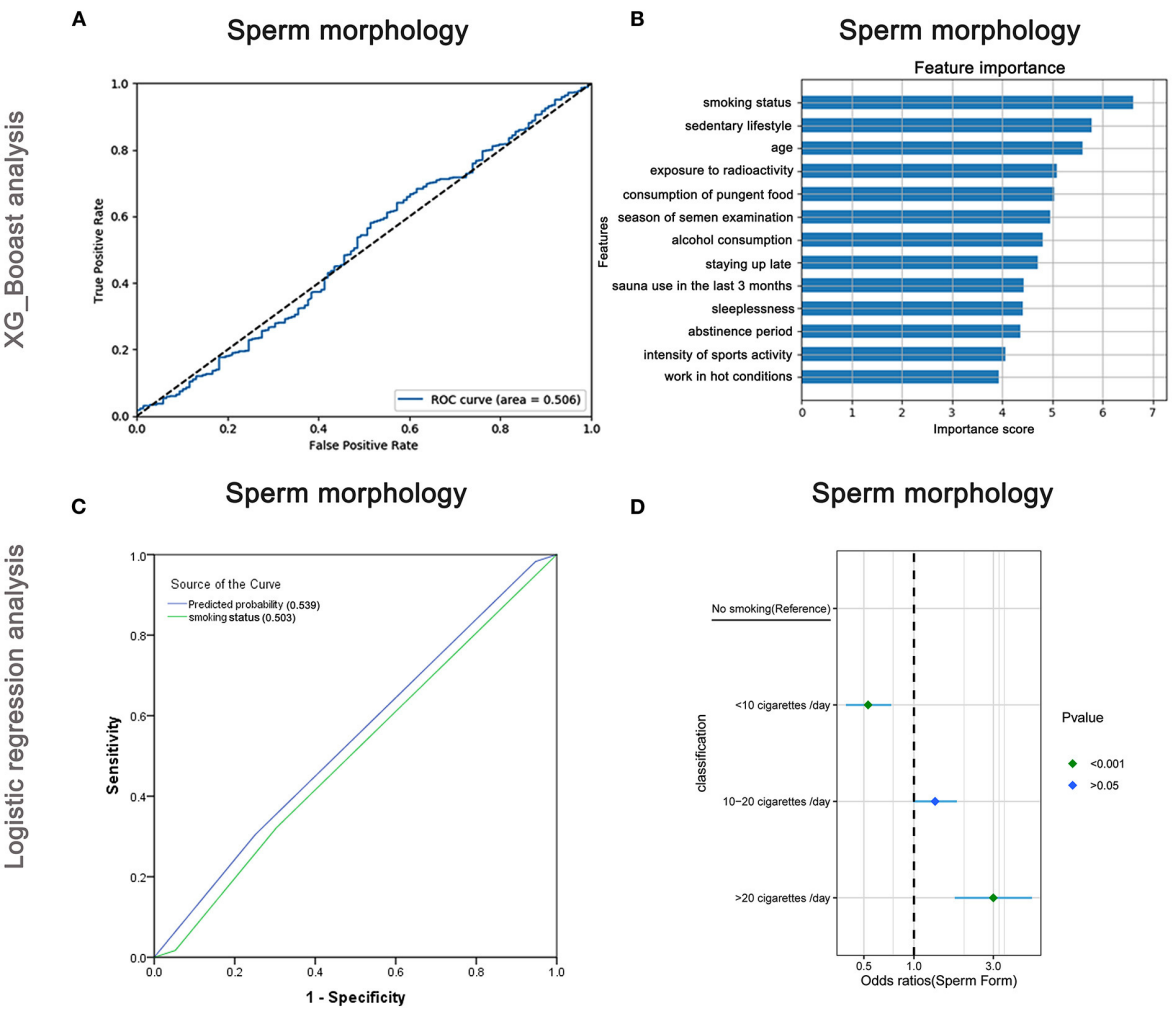
XGBoost and logistic regression analysis of the risk factors for sperm morphology. The ROC curve **(A)** and feature importance **(B)** analyzed by XGBoost and the ROC curve **(C)** and forest diagram showing significant risk factors **(D)** analyzed by logistic regression.

The AUC of the XGBoost model for sperm morphology was only 0.506 (Figure 7A), and the cross-validation showed that the AUC of the model was 0.520. The feature importance plot created using XGBoost showed that smoking status was the maximum important factor (Figure 7B). The AUCs of the logistic regression analyses (Figure 7C) revealed that smoking status was a predictive index for sperm morphology with a poor AUC (0.539), and the cross-validation based on the multivariate regression analysis showed that the AUC of the model was 0.543. As shown in Figure 7D and Supplementary Table 8, males who smoked more than 20 cigarettes/day were more likely to have abnormal sperm morphology than non-smokers (OR: 3.0, 95% CI: 1.76–5.12, *P* < 0.001), but the trend did not appear for males who smoked <10 cigarettes/day (OR: 0.53, 95% CI: 0.39–0.73, *P* < 0.001).

### Risk factors affecting DFI

**Figure 8:**
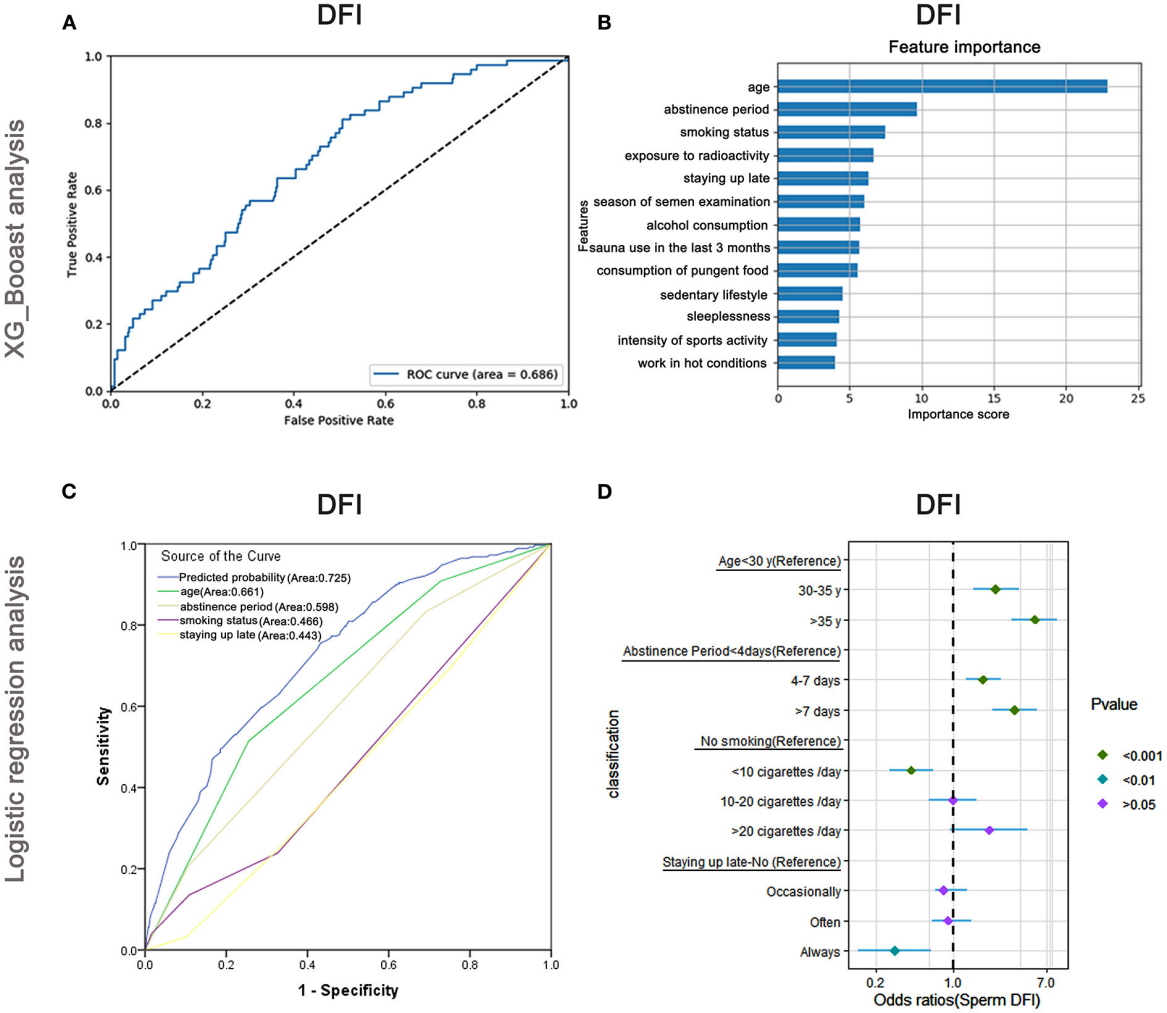
XGBoost and logistic regression analysis of the risk factors for the DNA fragmentation index (DFI). The ROC curve **(A)** and feature importance **(B)** analyzed by XGBoost and the ROC curve **(C)** and forest diagram showing significant risk factors **(D)** analyzed by logistic regression.

The AUC of the XGBoost model for DFI was 0.686 (Figure 8A), and the cross-validation showed that the AUC of the model was 0.697. The top three important features affecting total sperm vitality were age, abstinence period, and smoking status (Figure 8B). The AUCs of the logistic regression analyses (Figure 8C) revealed that age, abstinence period, smoking status, and staying up late were predictive markers of sperm DFI. The AUC of the combined marker (AUC = 0.725) was higher than that of the other individual markers (AUC = 0.661, 0.598, 0.466, and 0.443). The cross-validation based on the multivariate regression analysis showed that the AUC of the model was 0.648. The top-two odds ratios appeared for age and abstinence period (OR = 5.47 and 3.61), indicating their important predictive roles. Smoking status, the third important factor revealed by XGBoost, was also shown to significantly affect sperm DFI in regression analysis (Supplementary Table 9).

Besides, as shown in Figure 8D and Supplementary Table 9, older males (>35 years) and those maintaining abstinence for more than 7 days were more likely to have higher DFI (OR: 5.47, 95% CI: 3.41–8.76, *P* < 0.001 and OR: 3.61, 95% CI: 2.27–5.75, *P* < 0.001) than younger males (<30 years old) and those maintaining abstinence for <4 days, respectively. Males who smoked <10 cigarettes /day were less likely to have a high DFI (OR: 0.42, 95% CI: 0.27–0.66, *P* < 0.001) than non-smokers. Nevertheless, when they smoked more than 20 cigarettes/day, the odds ratio of having high a DFI increased (*P* > 0.05).

### Correlations between general information

Considering the close relationships among the variables, Spearman rank correlation analysis was performed. As shown in Supplementary Table 10, significant positive correlations were observed between sedentary lifestyle and age, staying up late, sleeplessness, consumption of pungent food, and exposure to radioactivity, with correlation coefficient (ICC) values of 0.043, 0.078, 0.056, 0.061, and 0.438, respectively. Meanwhile, sedentary lifestyles showed negative correlations with smoking status, intensity of sports activity, and working in hot conditions (ICC = −0.088, −0.119, and −0.134, respectively). Positive correlations were observed between staying up late and smoking status, alcohol consumption, sleeplessness, consumption of pungent food, sedentary lifestyle, working in hot conditions, sauna use in the last 3 months, and exposure to radioactivity (ICC = 0.185, 0.238, 0.310, 0.342, 0.078, 0.087, 0.067, and 0.034; *P* < 0.05), but staying up late showed negative correlations with age, abstinence period, and intensity of sports activity (ICC = −0.074, −0.055, −0.067; *P* < 0.05). Exposure to radioactivity showed positive correlations with staying up late, consumption of pungent food, and a sedentary lifestyle (ICC = 0.034, 0.046, and 0.438; *P* < 0.05), but showed negative correlations with smoking status, alcohol consumption, and working in hot conditions (ICC = −0.142, −0.028, and −0.109).

## Discussion

The factors influencing semen quality are complex. Several studies have reported that male age and environmental/lifestyle exposures, rather than the genetic problems, are primarily responsible for abnormal semen quality (1, 22, 23). Among these, lifestyle factors can be easily modified without medical interventions (24), and elucidate the lifestyle factors affecting semen quality can guide men to take appropriate measures in the preconception period. However, as described above, the lifestyles leading to abnormal semen quality have not been completely clarified, while the complexity of these data made related analysis difficult. In recent years, the wide application of AI provided a new method for related research (13).

Since the typical tabular data in our research were more suitable for the decision tree algorithm, and XGBoost is generally superior to other decision tree algorithms such as GBDT random forest and artificial neural network models in terms of predictive performance (25–27), we constructed a preliminary lifestyle- and general factor-based semen quality prediction model *via* machine learning with the XGBoost algorithm by using data collected from 5,109 healthy men. Furthermore, since the accuracy of machine learning algorithms may be impaired because of overfitting or insufficient data training (12, 28–30), we have applied logistic regression combined with cross-validation to verify the accuracy and the feasibility of machine learning-based prediction model.

After training the XGBoost with 13 potential affecting factors, the results showed that the AUCs of semen volume, sperm concentration, sperm progressive and total sperm motility, and DFI were 0.648, 0.661, 0.697, 0.660, and 0.686, respectively, which was consistent with the regression model and the subsequent cross-validation. In addition, the top two important factors affecting semen volume, sperm concentration, and the top three important factors affecting sperm motility and DFI indicated by the XGBoost were also revealed as predictive indices by regression analysis, indicating the promising predictive value of machine learning. However, both the XGBoost model and logistic regression assay as well as the following cross-validation based on sperm morphology showed poor predictive values (AUC = 0.506, 0.520, 0.539, and 0.543). We speculate that this could be because lifestyle-related factors have minimal influence on sperm morphology (31), which is primarily mediated by genetic factors (32). The XGBoost prediction model indicated that smoking status was the most important factor affecting the parameters of semen volume, sperm concentration, and motility and was the third important factor affecting DFI, and the results were verified by regression analysis. Many other studies have also indicated cigarette smoking has an overall negative effect on the semen parameters because the toxins originating from cigarette smoke can decrease sperm mitochondrial activity and damage the chromatin structure in human sperm (33–36). The regression assay further revealed that heavy smoking (>20 cigarettes/day) posed a harmful effect, which suggested that men of reproductive age men should give up heavy smoking first. However, it was interesting that mild (<10 cigarettes/day) smoking had positive consequents, which was partly consistent with the findings of Kemal and Adelusi et al. (37, 38). They found that smokers showed a higher percentage of rapidly progressive sperm. The possible reason for this result is that mild smoking could generate trace amounts of oxides, which are required to support both sperm motility and capacitation (39). Moreover, our results inevitably showed interference since many patients who smoke very occasionally (<1 smoke/day) were categorized into the mild smoking group. Further adjustment and improvement of questionnaire designs will be performed in the following research.

Furthermore, the abstinence period was the second-most important factor influencing semen volume, progressive sperm motility, and DFI. The regression analysis further showed that longer abstinence periods (>7 days) can help increase semen volume, but would hurt sperm motility and increase sperm DFI. Sperm motility has been shown to peak within 4 or 5 days of abstinence (40), and spermatozoa accumulating in the epididymis might react with oxygen and nitrogen species (ROS and RNS) during prolonged periods of ejaculatory abstinence (41). Thus, males should maintain a healthy rhythm of sex to ensure optimal semen quality.

Age is the primary risk factor affecting semen DFI and a secondary risk factor affecting semen density. The regression assay revealed that the sperm DFI was higher in elder men, and oxidative stress damage might be one of the mechanisms underlying this finding (42–44). Meanwhile, the semen volume decreased and sperm density increased with increasing age, which might be attributable to prostate atrophy. Increased age is known to be associated with genome-wide mutations, DFI, and chromatin integrity (45), and high sperm DFI is associated with spontaneous abortion (46, 47). Thus, men should be to encouraged to have children early.

In addition, other factors explored in this study, except sauna use in the last 3 months and sleeplessness, influenced semen parameters to some extent. Curiously, unlike published research stating that a sedentary lifestyle or playing computer games adversely affected sperm motility (48), our regression analysis revealed that individuals with predominantly sedentary lifestyles were less likely to have lower semen volume and those exposed to computer radiation constantly were less likely to have lower sperm motility. Moreover, men who slept late were less likely to have a high DFI. However, the correlation analysis (please see the Supplementary Table 10) revealed that sedentary lifestyles and prolonged computer usage showed negative correlations with smoking status and late sleeping hours showed a negative correlation with age, which may be one reason for the confusing results.

Our study had some limitations. First, all data were collected from our own center without external validation, and we recruited patients receiving assisted fertility guidance or treatment, which may not fully represent the general population. Second, most lifestyle factors were self-reported and were subjective constructs in this research. Moreover, the stages of changes in most lifestyle factors could not be precisely delineated, and the valid data sample was not large enough to obtain precise predictions. Under the influence of the various factors described above, the current results showed that the XGBoost Algorithm had no obvious advantage over logistic regression. However, considering its benefits of allowing flexible analyses of relationships among predictor variables and outcomes in large datasets as well as the easy online updates in the prediction system, its implementation into the clinical workflow can be advantageous. We believe that the XGBoost will have promising predictive value and guiding significance after enlarging the data sample size and data feature dimensions as well adding information-based data extraction methods.

## Conclusion

In summary, the preliminary model for predicting semen quality using lifestyle factors that was developed with the XGBoost algorithm had the potential to undergo further optimization with larger training data. In addition, the model suggested that smoking status, abstinence period, and age were important factors affecting semen quality parameters.

## Data availability statement

The original contributions presented in the study are included in the article/Supplementary material, further inquiries can be directed to the corresponding authors.

## Ethics statement

This study was approved by the Ethics Committee of Ruijin Hospital, School of Medicine, Shanghai Jiao Tong University (No. 2019-185), and all participants recruited signed informed consent forms.

## Author contributions

BX, AZ, and YG designed the study and headed the interdisciplinary exchange. TY, YG, and HH performed machine learning. JL and MZ undertook the statistical analyses. WF examined semen parameters. MZ, BX, TY, and AZ collected the data and drafted the manuscript. All authors contributed to the article and approved the submitted version.

## Funding

This work was supported by grants from the Shanghai Jiao Tong University Medicine-Engineering Fund (Grant Number YG2017MS57), the National Natural Science Foundation of China (Grant Numbers 82071712 and 81771656), the Shanghai Medicine and Health Development Foundation (Grant Number SHWJRS(2021)-99 to BX), and the Guangci Clinical New Technology Sailing Plan of Ruijin Hospital (Grant Number GCQH-2021-07).

## Conflict of interest

Author TY is employed by Shanghai National Engineering Research Center of Digital Television Co., Ltd. This work is not funded by Shanghai National Engineering Research Center of Digital Television Co., Ltd. The remaining authors declare that the research was conducted in the absence of any commercial or financial relationships that could be construed as a potential conflict of interest.

## Publisher's note

All claims expressed in this article are solely those of the authors and do not necessarily represent those of their affiliated organizations, or those of the publisher, the editors and the reviewers. Any product that may be evaluated in this article, or claim that may be made by its manufacturer, is not guaranteed or endorsed by the publisher.

## References

[1] Virtanen HE Jørgensen N Toppari J. Semen quality in the 21 century. Nat Rev Urol. (2017) 14:120–30. 10.1038/nrurol.2016.261 28050014

[2] Agarwal A Baskaran S Parekh N Cho C-L Henkel R Vij S. Male infertility. Lancet. (2021) 397:319–33. 10.1016/S0140-6736(20)32667-2 33308486

[3] Huang C Li B Xu K Liu D Hu J Yang Y. Decline in semen quality among 30,636 young Chinese men from 2001 to 2015. Fertility Sterility. (2017) 107:35. 10.1016/j.fertnstert.2016.09.035 27793371

[4] Wang L Zhang L Song X-H Zhang H-B Xu C-Y Chen Z-J. Decline of semen quality among Chinese sperm bank donors within 7 years (2008-2014). Asian J Androl. (2017) 19:521–5. 10.4103/1008-682X.179533 27345004 PMC5566843

[5] Verze P Cai T Lorenzetti S. The role of the prostate in male fertility, health and disease. Nat Rev Urol. (2016) 13:379–86. 10.1038/nrurol.2016.89 27245504

[6] Skakkebaek NE Rajpert-De Meyts E Buck Louis GM Toppari J Andersson A-M Eisenberg ML. Male reproductive disorders and fertility trends: influences of environment and genetic susceptibility. Physiol Rev. (2016) 96:55–97. 10.1152/physrev.00017.2015 26582516 PMC4698396

[7] Krausz C Riera-Escamilla A. Genetics of male infertility. Nat Rev Urol. (2018) 15:369–84. 10.1038/s41585-018-0003-3 29622783

[8] Punab M Poolamets O Paju P Vihljajev V Pomm K Ladva R. Causes of male infertility: a 9-year prospective monocentre study on 1737 patients with reduced total sperm counts. Hum Reprod. (2017) 32:18–31. 10.1093/humrep/dew284 27864361 PMC5165077

[9] de Kretser DM. Male infertility. Lancet. (1997) 349:787–90. 10.1016/S0140-6736(96)08341-9 9074589

[10] Husáková P Ulcová-Gallová Z Bibková K Micanová Z. Semen quality of Pilsner University students. Cas Lek Cesk. (2008) 147:85–8. 18383958

[11] Giwercman A Giwercman YL. Environmental factors and testicular function. Best Pract Res Clin Endocrinol Metab. (2011) 25:391–402. 10.1016/j.beem.2010.09.011 21397206

[12] Goldstein BA Navar AM Carter RE. Moving beyond regression techniques in cardiovascular risk prediction: applying machine learning to address analytic challenges. Eur Heart J. (2017) 38:1805–14. 10.1093/eurheartj/ehw302 27436868 PMC5837244

[13] Wang R Pan W Jin L Li Y Geng Y Gao C. Artificial intelligence in reproductive medicine. Reproduction. (2019) 158:R139–54. 10.1530/REP-18-0523 30970326 PMC6733338

[14] Topol EJ. High-performance medicine: the convergence of human and artificial intelligence. Nat Med. (2019) 25:44–56. 10.1038/s41591-018-0300-7 30617339

[15] Hamet P Tremblay J. Artificial intelligence in medicine. Metabolism. (2017) 69S:S36–40. 10.1016/j.metabol.2017.01.011 28126242

[16] Riegler MA Stensen MH Witczak O Andersen JM Hicks SA Hammer HL. Artificial intelligence in the fertility clinic: status, pitfalls and possibilities. Hum Reprod. (2021) 36:2429–42. 10.1093/humrep/deab168 34324672

[17] Girela JL Gil D Johnsson M Gomez-Torres MJ De Juan J. Semen parameters can be predicted from environmental factors and lifestyle using artificial intelligence methods. Biol Reprod. (2013) 88:99. 10.1095/biolreprod.112.104653 23446456

[18] Sahoo AJ Kumar Y. Seminal quality prediction using data mining methods. Technol Health Care. (2014) 22:531–45. 10.3233/THC-140816 24898862

[19] Lagerros YT Mucci LA Bellocco R Nyrén O Bälter O Bälter KA. Validity and reliability of self-reported total energy expenditure using a novel instrument. Eur J Epidemiol. (2006) 21:227–36. 10.1007/s10654-006-0013-y 16547838

[20] Cooper TG Noonan E von Eckardstein S Auger J Baker HWG Behre HM. World Health Organization reference values for human semen characteristics. Hum Reprod Update. (2010) 16:231–45. 10.1093/humupd/dmp048 19934213

[21] Le MT Nguyen TAT Nguyen HTT Nguyen TTT Nguyen VT Le DD. Does sperm DNA fragmentation correlate with semen parameters? Reprod Med Biol. (2019) 18:390–6. 10.1002/rmb2.12297 31607800 PMC6780033

[22] Tournaye H Krausz C Oates RD. Concepts in diagnosis and therapy for male reproductive impairment. Lancet Diabetes Endocrinol. (2017) 5:554–64. 10.1016/S2213-8587(16)30043-2 27395770

[23] Ilacqua A Izzo G Emerenziani GP Baldari C Aversa A. Lifestyle and fertility: the influence of stress and quality of life on male fertility. Reprod Biol Endocrinol. (2018) 16:115. 10.1186/s12958-018-0436-9 30474562 PMC6260894

[24] Mulder M Ranchor AV Sanderman R Bouma J van den Heuvel WJ. The stability of lifestyle behaviour. Int J Epidemiol. (1998) 27:199–207. 10.1093/ije/27.2.199 9602399

[25] Yue S Li S Huang X Liu J Hou X Zhao Y. Machine learning for the prediction of acute kidney injury in patients with sepsis. J Transl Med. (2022) 20:215. 10.1186/s12967-022-03364-0 35562803 PMC9101823

[26] Zhu Y Zhang J Wang G Yao R Ren C Chen G. Machine learning prediction models for mechanically ventilated patients: analyses of the MIMIC-III database. Front Med. (2021) 8:662340. 10.3389/fmed.2021.662340 34277655 PMC8280779

[27] Lee H-C Yoon SB Yang S-M Kim WH Ryu H-G Jung C-W. Prediction of acute kidney injury after liver transplantation: machine learning approaches vs. logistic regression model. J Clin Med. (2018) 7:428. 10.3390/jcm7110428 30413107 PMC6262324

[28] Rajkomar A Dean J Kohane I. Machine learning in medicine. N Engl J Med. (2019) 380:1347–58. 10.1056/NEJMra1814259 30943338

[29] Davenport T Kalakota R. The potential for artificial intelligence in healthcare. Future Healthc J. (2019) 6:94–8. 10.7861/futurehosp.6-2-94 31363513 PMC6616181

[30] Collins GS Moons KGM. Reporting of artificial intelligence prediction models. Lancet. (2019) 393:1577–9. 10.1016/S0140-6736(19)30037-6 31007185

[31] Pacey AA Povey AC Clyma JA McNamee R Moore HD Baillie H. Modifiable and non-modifiable risk factors for poor sperm morphology. Hum Reprod. (2014) 29:1629–36. 10.1093/humrep/deu116 24899128

[32] Ray PF Toure A Metzler-Guillemain C Mitchell MJ Arnoult C Coutton C. Genetic abnormalities leading to qualitative defects of sperm morphology or function. Clin Genet. (2017) 91:217–32. 10.1111/cge.12905 27779748

[33] Mostafa RM Nasrallah YS Hassan MM Farrag AF Majzoub A Agarwal A. The effect of cigarette smoking on human seminal parameters, sperm chromatin structure and condensation. Andrologia. (2018) 50:12910. 10.1111/and.12910 29124782

[34] Mohamad Al-Ali B Eredics K. Synergistic effects of cigarette smoking and varicocele on semen parameters in 715 patients. Wien Klin Wochenschr. (2017) 129:482–6. 10.1007/s00508-017-1199-6 28439698

[35] Keskin MZ Budak S Gubari S Durmaz K Yoldas M Celik O. Do cigarette and alcohol affect semen analysis? Arch Ital Urol Androl. (2016) 88:56–9. 10.4081/aiua.2016.1.56 27072177

[36] Sharma R Harlev A Agarwal A Esteves SC. Cigarette smoking and semen quality: a new meta-analysis examining the effect of the 2010. World Health Organization Laboratory Methods for the Examination of Human Semen. Eur Urol. (2016) 70:635–45. 10.1016/j.eururo.2016.04.010 27113031

[37] Ozgur K Isikoglu M Seleker M Donmez L. Semen quality of smoking and non-smoking men in infertile couples in a Turkish population. Arch Gynecol Obstet. (2005) 271:109–12. 10.1007/s00404-003-0572-z 14685893

[38] Adelusi B al-Twaijiri MH al-Meshari A Kangave D al-Nuaim LA Younnus B. Correlation of smoking and coffee drinking with sperm progressive motility in infertile males. Afr J Med Med Sci. (1998) 27:47–50. 10456129

[39] O'Flaherty CM Beorlegui NB Beconi MT. Lactate dehydrogenase-C4 is involved in heparin- and NADH-dependent bovine sperm capacitation. Andrologia. (2002) 34:91–7. 10.1046/j.0303-4569.2001.00481.x 11966575

[40] Hanson BM Aston KI Jenkins TG Carrell DT Hotaling JM. The impact of ejaculatory abstinence on semen analysis parameters: a systematic review. J Assist Reprod Genet. (2018) 35:213–20. 10.1007/s10815-017-1086-0 29143943 PMC5845044

[41] Borges E Braga DPAF Zanetti BF Iaconelli A Setti AS. Revisiting the impact of ejaculatory abstinence on semen quality and intracytoplasmic sperm injection outcomes. Andrology. (2019) 7:213–9. 10.1111/andr.12572 30570220

[42] Johnson SL Dunleavy J Gemmell NJ Nakagawa S. Consistent age-dependent declines in human semen quality: a systematic review and meta-analysis. Ageing Res Rev. (2015) 19:22–33. 10.1016/j.arr.2014.10.007 25462195

[43] Eskenazi B Wyrobek AJ Sloter E Kidd SA Moore L Young S. The association of age and semen quality in healthy men. Hum Reprod. (2003) 18:447–54. 10.1093/humrep/deg107 12571189

[44] Brahem S Mehdi M Elghezal H Saad A. The effects of male aging on semen quality, sperm DNA fragmentation and chromosomal abnormalities in an infertile population. J Assist Reprod Genet. (2011) 28:425–32. 10.1007/s10815-011-9537-5 21287403 PMC3151353

[45] Kong A Frigge ML Masson G Besenbacher S Sulem P Magnusson G. Rate of de novo mutations and the importance of father's age to disease risk. Nature. (2012) 488:471–5. 10.1038/nature11396 22914163 PMC3548427

[46] Lin M-H Kuo-Kuang Lee R Li S-H Lu C-H Sun F-J Hwu Y-M. Sperm chromatin structure assay parameters are not related to fertilization rates, embryo quality, and pregnancy rates in *in vitro* fertilization and intracytoplasmic sperm injection, but might be related to spontaneous abortion rates. Fertil Steril. (2008) 90:352–9. 10.1016/j.fertnstert.2007.06.018 17904130

[47] McQueen DB Zhang J Robins JC. Sperm DNA fragmentation and recurrent pregnancy loss: a systematic review and meta-analysis. Fertility Sterility. (2019) 112:54–60. 10.1016/j.fertnstert.2019.03.003 31056315

[48] Gaskins AJ Mendiola J Afeiche M Jørgensen N Swan SH Chavarro JE. Physical activity and television watching in relation to semen quality in young men. Br J Sports Med. (2015) 49:265–70. 10.1136/bjsports-2012-091644 23380634 PMC3868632

