# Supplementary material for: Preliminary prediction of semen quality based on modifiable lifestyle factors by using the XGBoost algorithm
Source: Front Med (Lausanne). 2022 Sep 13;9:811890. doi: 10.3389/fmed.2022.811890 (PMC9514383; doi:10.3389/fmed.2022.811890)
Supplement: Supplementary file 1 [file Table_1.docx]

### Supplementary Table 1. Questionnaire and the classification criteria.

| Items | Fill the following blanks or select the options | | | | | | | | The criterion of categorical variable split | | | | | |
| --- | --- | --- | --- | --- | --- | --- | --- | --- | --- | --- | --- | --- | --- | --- |
| Date (Day/Month/Year) |  | | | | | | | | Spring (March 21st-Jun 20st) | Summer (June 21st-Septemnber 22st) | Autumn (September 23st-December 21st) | Winter (December 22st-March 20st) | | |
| Age (years) |  | | | | | | | | < 30 | 30-35 | > 35 | | | |
| Abstinence period (day) |  | | | | | | | | < 4 | 4-7 | > 7 | | | |
| Smoking in the latest two years (cigarettes /day) |  | | | | | | | | 0 | <9 | 10-20 | | >20 | |
| Alcohol consumption per day in the latest two years | Light beer (ethanol:<4% vol, 330ml/botton or can) | | Regular beer (ethanol:≥4% vol,330ml/botton or can) | Wine (4 ounce glass) | | Spirits (ethanol:<30% vol,10ml/glass) | Spirits (ethanol:30-50% vol,10ml/glass) | Liquor (ethanol:>50% vol, 10ml/glass) | 0 | < 9.9g/day | 9.9-19 g/day | | >19 g/day | |
|  |  |  | |  | |  |  |  |  |  |  |  |  |  |
| Bedtimes (PM) |  | | | | | | | | < 10 PM | 10-10:59 PM | 11-12 PM | | >12PM | |
| Sleeplessness (score of ISI) | Please fill the scale of Insomnia Severity Index (ISI) in supplementary table 2. | | | | | | | | 0-7 | 8-14 | 15-21 | | 22-28 | |
| Pungent-flavor food consumption | 🞏Never; 🞏<1 day per week; 🞏1-5 days a week; 🞏> 5 days a week | | | | | | | | Never | <1 day per week (occasionally) | 1-5 days a week (often) | | > 5 days a week (aways) | |
| Sports # | Please fill the Physical Activity Questionnaires in supplementary table 3. | | | | | | | | ≤1.99METs | 2-2.99METs | 3-3.99METs | | 4-5.99METs | ≥6 METs |
| Sedentary (h/day) |  | | | | | | | | < 5 | ≥5 | | | | |
| Does your work under high environment temperature exposure (WBGT≥ 25℃)? | 🞏 No | | | | Yes | | | | No | Yes | | | | |
|  |  |  |  |  | I’m a (🞏 outdoor construction worker; 🞏steelworker; 🞏truck driver; 🞏others____), and I have received the high-temperature allowance in the last three months. | | | |  |  |  |  |  |  |
| Sauna usage in the latest 3 months | 🞏 No | | | | 🞏 Yes | | | | No | Yes | | | | |
| Do you often under electromagnetic radiation exposure? | 🞏 No | | | | Yes | | | | No | Computer | Radio | | Others | |
|  |  |  |  |  | 🞏computer; 🞏Radio; 🞏others___ | | | |  |  |  |  |  |  |

# The scoring criteria are as described in the article (PMID: 16547838)
