# Supplementary material for: Preliminary prediction of semen quality based on modifiable lifestyle factors by using the XGBoost algorithm
Source: Front Med (Lausanne). 2022 Sep 13;9:811890. doi: 10.3389/fmed.2022.811890 (PMC9514383; doi:10.3389/fmed.2022.811890)
Supplement: Supplementary file 2 [file Table_2.docx]

**Supplementary Table 2.** Athens Insomnia Scale

ID:__________ Age:_________ Sex:_________ Date:________

Instructions: This scale is intended to record your own assessment of any sleep difficulty you might have experienced. Please, check (by circling the appropriate number) the items below to indicate your estimate of any difficulty, provided that it occurred at least three times per week during the last month

Sleep induction (time it takes you to fall asleep after turning-off the lights)

0: No problem 1: Slightly delayed 2: Markedly delayed 3: Very delayed or did not sleep at all

Awakenings during the night

0: No problem 1: Minor problem 2: Considerable problem 3: Serious problem or did not sleep at all

Final awakening earlier than desired

0: Not earlier 1: A little earlier 2: Markedly earlier 3: Much earlier or did not sleep at all

Total sleep duration

0: Sufficient 1: Slightly insufficient 2: Markedly insufficient 3: Very insufficient or did not sleep at all

Overall quality of sleep (no matter how long you slept)

0: Satisfactory 1: Slightly unsatisfactory 2: Markedly unsatisfactory 3: Very unsatisfactory or did not sleep at all

Sense of well-being during the day

0: Normal 1: Slightly decreased 2: Markedly decreased 3: Very decreased

Functioning (physical and mental) during the day

0: Normal 1: Slightly decreased 2: Markedly decreased 3: Very decreased

Sleepiness during the day

0: None 1: Mild 2: Considerable 3: Intense
