# Supplementary material for: Preliminary prediction of semen quality based on modifiable lifestyle factors by using the XGBoost algorithm
Source: Front Med (Lausanne). 2022 Sep 13;9:811890. doi: 10.3389/fmed.2022.811890 (PMC9514383; doi:10.3389/fmed.2022.811890)
Supplement: Supplementary file 3 [file Table_3.docx]

**Supplementary Table 3.** Physical Activity Questionnaires

ID:__________ Age:_________ Sex:_________ Date:________

Instructions: How much time a day do you spend doing activities as demanding as:

A for example sleeping, lying quietly in bed

Hours ___ Minutes ___

B for example sitting - bathing, quietly listening to music, watching television, etc.

Hours ___ Minutes ___

C for example sitting – light office work, knitting, sewing, meetings, etc.

Hours ___ Minutes ___

D for example making bed, ironing, washing dishes, etc.

Hours ___ Minutes ___

E for example bowling, driving bus/tractor, automobile repair, public square dancing, etc.

Hours ___ Minutes ___

F for example walking briskly, electro-mobile riding, sweeping, sidewalk, etc.

Hours ___ Minutes ___

G for example painting outside house, carrying and stacking wood, etc.

Hours ___ Minutes ___

H for example construction work, mowing lawn with hand mower, etc.

Hours ___ Minutes ___

I more effort than level H

Hours ___ Minutes ___
