# Supplementary material for: Preliminary prediction of semen quality based on modifiable lifestyle factors by using the XGBoost algorithm
Source: Front Med (Lausanne). 2022 Sep 13;9:811890. doi: 10.3389/fmed.2022.811890 (PMC9514383; doi:10.3389/fmed.2022.811890)
Supplement: Supplementary file 4 [file Table_4.docx]

**Supplementary Table 4.** Univariate and multivariate analyses of semen volume

| Variable | Controls |  | Univariate analysis | | |  |  | Multivariate analysis | | |  |
| --- | --- | --- | --- | --- | --- | --- | --- | --- | --- | --- | --- |
|  |  | β | OR | 95%CI | *p-*value |  | β | OR | 95%CI | *p-*value |  |
| Season of semen examination | Spring | Reference |  |  |  |  |  |  |  |  |  |
|  | Summer | -0.128 | 0.88 | 0.67-1.15 | 0.3487 |  |  |  |  |  |  |
|  | Autumn | -0.344 | 0.71 | 0.52-0.97 | 0.0304 |  |  |  |  |  |  |
|  | Winter | 0.024 | 1.02 | 0.80-1.31 | 0.8491 |  |  |  |  |  |  |
| Age (years) | < 30 | Reference |  |  |  |  | Reference |  |  |  |  |
|  | 30-35 | -2.455 | 0.95 | 0.75-1.21 | 0.6770 |  | 0.046 | 1.05 | 0.82-1.34 | 0.7134 |  |
|  | > 35 | -0.051 | 1.53 | 1.19-1.98 | 0.0010 |  | 0.518 | 1.68 | 1.29-2.18 | 0.0001 |  |
| Abstinence period (days) | <4 | Reference |  |  |  |  | Reference |  |  |  |  |
|  | 4-7 | -0.719 | 0.49 | 0.40-0.60 | <.0001 |  | -0.692 | 0.5 | 0.40-0.62 | <.0001 |  |
|  | >7 | -0.426 | 0.65 | 0.48-0.90 | 0.0082 |  | -0.458 | 0.63 | 0.46-0.87 | 0.0054 |  |
| Smoking status (cigarettes /day) | 0 | Reference |  |  |  |  | Reference |  |  |  |  |
|  | <10 | -0.384 | 0.68 | 0.49-0.94 | 0.0211 |  | -0.404 | 0.67 | 0.48-0.93 | 0.0161 |  |
|  | 10-20 | -0.221 | 0.8 | 0.56-1.15 | 0.2328 |  | -0.304 | 0.74 | 0.51-1.06 | 0.1034 |  |
|  | >20 | 1.611 | 5.01 | 3.644-6.882 | <.0001 |  | 1.546 | 4.69 | 3.39-6.49 | <.0001 |  |
| Alcohol consumption (g/day) | 0 | Reference |  |  |  |  |  |  |  |  |  |
|  | < 9.9 | 0.002 | 1 | 0.82-1.23 | 0.5627 |  |  |  |  |  |  |
|  | 10-18.9 | 0.347 | 1.42 | 0.91-2.21 | 0.6752 |  |  |  |  |  |  |
|  | >19 | 3.072 | 21.57 | 1.95-238.82 | 0.2424 |  |  |  |  |  |  |
| Staying_up_late | never | Reference |  |  |  |  |  |  |  |  |  |
|  | Occasionally | -0.074 | 0.93 | 0.72-1.19 | 0.5627 |  |  |  |  |  |  |
|  | Often | 0.06 | 1.06 | 0.80-1.40 | 0.6752 |  |  |  |  |  |  |
|  | Always | 0.207 | 1.23 | 0.87-1.74 | 0.2424 |  |  |  |  |  |  |
| Sleeplessness | never | Reference |  |  |  |  |  |  |  |  |  |
|  | Occasionally | 0.096 | 1.1 | 0.89-1.36 | 0.3776 |  |  |  |  |  |  |
|  | Often | 0.262 | 1.3 | 0.93-1.82 | 0.1295 |  |  |  |  |  |  |
|  | Always | -0.211 | 0.81 | 0.29-2.25 | 0.6859 |  |  |  |  |  |  |
| Consumption of pungent food | never | Reference |  |  |  |  |  |  |  |  |  |
|  | Occasionally | -0.152 | 0.86 | 0.66-1.12 | 0.2683 |  |  |  |  |  |  |
|  | Often | -0.005 | 1 | 0.74-1.35 | 0.9761 |  |  |  |  |  |  |
|  | Always | -0.097 | 0.91 | 0.53-1.55 | 0.722 |  |  |  |  |  |  |
| Intensity of sports activity (times/week) | 0 | Reference |  |  |  |  |  |  |  |  |  |
|  | <1 | -0.171 | 0.84 | 0.61-1.17 | 0.3028 |  |  |  |  |  |  |
|  | 2-3 | -0.201 | 0.82 | 0.59-1.13 | 0.2279 |  |  |  |  |  |  |
|  | 4-5 | -0.002 | 1 | 0.62-1.61 | 0.9919 |  |  |  |  |  |  |
|  | >5 | 0.282 | 1.33 | 0.60-2.94 | 0.4868 |  |  |  |  |  |  |
| Sedentary lifestyle | No | Reference |  |  |  |  | Reference |  |  |  |  |
|  | Yes | -0.216 | 0.81 | 0.66-0.99 | 0.0416 |  | -0.215 | 0.81 | 0.65-1.00 | 0.0486 |  |
| Work in hot conditions | No | Reference |  |  |  |  |  |  |  |  |  |
|  | Yes | 0.123 | 1.13 | 0.74-1.73 | 0.5705 |  |  |  |  |  |  |
| Sauna use in the last 3 months | No | Reference |  |  |  |  |  |  |  |  |  |
|  | Yes | 0.444 | 1.56 | 0.86-2.81 | 0.1405 |  |  |  |  |  |  |
| Exposure to radioactivity (Source) | None | Reference |  |  |  |  |  |  |  |  |  |
|  | Computer | -0.139 | 0.87 | 0.70-1.08 | 0.2057 |  |  |  |  |  |  |
|  | Rays | 0.869 | 2.39 | 0.50-11.35 | 0.2746 |  |  |  |  |  |  |
|  | Others | -1.209 | 0.3 | 0.04-2.20 | 0.2354 |  |  |  |  |  |  |
