# Supplementary material for: Preliminary prediction of semen quality based on modifiable lifestyle factors by using the XGBoost algorithm
Source: Front Med (Lausanne). 2022 Sep 13;9:811890. doi: 10.3389/fmed.2022.811890 (PMC9514383; doi:10.3389/fmed.2022.811890)
Supplement: Supplementary file 5 [file Table_5.docx]

**Supplementary Table 5.** Univariate and multivariate analyses of sperm concentration

| Variable | Controls |  | Univariate analysis | | |  |  | Multivariate analysis | | |
| --- | --- | --- | --- | --- | --- | --- | --- | --- | --- | --- |
|  |  | β | OR | 95%CI | *p-*value |  | β | OR | 95%CI | *p-*value |
| Season of semen examination | Spring | Reference |  |  |  |  | Reference |  |  |  |
|  | Summer | 0.124 | 1.13 | 0.90-1.43 | 0.2985 |  |  |  |  |  |
|  | Autumn | 0.13 | 1.14 | 0.89-1.47 | 0.3129 |  |  |  |  |  |
|  | Winter | 0.176 | 1.19 | 0.96-1.49 | 0.1150 |  |  |  |  |  |
| Age (years) | < 30 | Reference |  |  |  |  | Reference |  |  |  |
|  | 30-35 | -0.458 | 0.63 | 0.52-0.77 | <.0001 |  | -0.418 | 0.66 | 0.54-0.80 | <.0001 |
|  | > 35 | -0.338 | 0.71 | 0.57-0.89 | 0.0028 |  | -0.331 | 0.72 | 0.57-0.91 | 0.0052 |
| Abstinence period (days) | <4 | Reference |  |  |  |  |  |  |  |  |
|  | 4-7 | -0.047 | 0.96 | 0.79-1.16 | 0.6328 |  |  |  |  |  |
|  | >7 | -0.043 | 0.96 | 0.72-1.27 | 0.7670 |  |  |  |  |  |
| Smoking status (cigarettes /day) | 0 | Reference |  |  |  |  | Reference |  |  |  |
|  | <10 | -1.982 | 0.14 | 0.08-0.24 | <.0001 |  | -2.08 | 0.13 | 0.07-0.22 | <.0001 |
|  | 10-20 | 0.277 | 1.32 | 1.02-1.71 | 0.0367 |  | 0.22 | 1.24 | 0.95-1.62 | 0.1079 |
|  | >20 | 2.035 | 7.65 | 5.72-10.24 | <.0001 |  | 1.94 | 6.97 | 5.18-9.36 | <.0001 |
| Alcohol consumption (g/day) | 0 | Reference |  |  |  |  |  |  |  |  |
|  | < 9.9 | -0.052 | 0.95 | 0.80-1.13 | 0.5546 |  |  |  |  |  |
|  | 10-18.9 | -0.002 | 1 | 0.65-1.53 | 0.9940 |  |  |  |  |  |
|  | >19 | 1.246 | 3.48 | 0.31-38.43 | 0.3096 |  |  |  |  |  |
| Staying_up_late | never | Reference |  |  |  |  |  |  |  |  |
|  | Occasionally | 0.069 | 1.07 | 0.87-1.33 | 0.5302 |  |  |  |  |  |
|  | Often | -0.016 | 0.99 | 0.77-1.26 | 0.9022 |  |  |  |  |  |
|  | Always | 0.348 | 1.42 | 1.05-1.90 | 0.0209 |  |  |  |  |  |
| Sleeplessness | never | Reference |  |  |  |  |  |  |  |  |
|  | Occasionally | 0.102 | 1.11 | 0.92-1.33 | 0.2725 |  |  |  |  |  |
|  | Often | 0.216 | 1.24 | 0.92-1.67 | 0.1521 |  |  |  |  |  |
|  | Always | -0.168 | 0.85 | 0.36-1.98 | 0.6982 |  |  |  |  |  |
| Consumption of pungent food | never | Reference |  |  |  |  | Reference |  |  |  |
|  | Occasionally | -0.24 | 0.79 | 0.63-0.99 | 0.0409 |  | -0.26 | 0.77 | 0.61-0.98 | 0.0321 |
|  | Often | 0.045 | 1.05 | 0.81-1.35 | 0.7267 |  | 0.03 | 1.03 | 0.79-1.34 | 0.8409 |
|  | Always | -0.072 | 0.93 | 0.59-1.46 | 0.7537 |  | 0.07 | 1.07 | 0.66-1.74 | 0.7851 |
| Intensity of sports activity (times/week) | 0 | Reference |  |  |  |  |  |  |  |  |
|  | <1 | -0.57 | 0.57 | 0.44-0.73 | <.0001 |  | -0.54 | 0.59 | 0.44-0.77 | 0.0001 |
|  | 2-3 | -0.561 | 0.57 | 0.44-0.74 | <.0001 |  | -0.51 | 0.60 | 0.46-0.79 | 0.0003 |
|  | 4-5 | -0.224 | 0.8 | 0.54-1.18 | 0.2558 |  | -0.22 | 0.80 | 0.53-1.20 | 0.2832 |
|  | >5 | -0.441 | 0.64 | 0.30-1.39 | 0.2638 |  | -0.36 | 0.70 | 0.31-1.57 | 0.3808 |
| Sedentary lifestyle | No | Reference |  |  |  |  |  |  |  |  |
|  | Yes | -0.185 | 0.83 | 0.70-0.99 | 0.0422 |  |  |  |  |  |
| Work in hot conditions | No | Reference |  |  |  |  |  |  |  |  |
|  | Yes | 0.123 | 1.13 | 0.74-1.73 | 0.5705 |  |  |  |  |  |
| Sauna use in the last 3 months | No | Reference |  |  |  |  |  |  |  |  |
|  | Yes | 0.444 | 1.56 | 0.86-2.81 | 0.1405 |  |  |  |  |  |
| Exposure to radioactivity (Source) | None | Reference |  |  |  |  |  |  |  |  |
|  | Computer | -0.139 | 0.87 | 0.70-1.08 | 0.2057 |  |  |  |  |  |
|  | Radio | 0.869 | 2.39 | 0.50-11.35 | 0.2746 |  |  |  |  |  |
|  | Others | -1.209 | 0.3 | 0.04-2.20 | 0.2354 |  |  |  |  |  |
