# Supplementary material for: Preliminary prediction of semen quality based on modifiable lifestyle factors by using the XGBoost algorithm
Source: Front Med (Lausanne). 2022 Sep 13;9:811890. doi: 10.3389/fmed.2022.811890 (PMC9514383; doi:10.3389/fmed.2022.811890)
Supplement: Supplementary file 6 [file Table_6.docx]

**Supplementary Table 6.** Univariate and multivariate analyses of progressive sperm motility

| Variable | Controls |  | Univariate analysis | | |  |  | Multivariate analysis | | |
| --- | --- | --- | --- | --- | --- | --- | --- | --- | --- | --- |
|  |  | β | OR | 95%CI | *p-*value |  | β | OR | 95%CI | *p-*value |
| Season of semen examination | Spring | Reference |  |  |  |  | Reference |  |  |  |
|  | Summer | 0.108 | 1.12 | 0.95-1.32 | 0.2123 |  | 0.066 | 1.07 | 0.89-1.28 | 0.4772 |
|  | Autumn | -0.251 | 0.78 | 0.64-0.95 | 0.0118 |  | -0.271 | 0.76 | 0.62-0.94 | 0.0109 |
|  | Winter | 0.1035 | 1.11 | 0.94-1.30 | 0.2074 |  | 0.093 | 1.097 | 0.92-1.30 | 0.2911 |
| Age (years) | < 30 | Reference |  |  |  |  | Reference |  |  |  |
|  | 30-35 | 0.076 | 0.89 | 0.77-1.03 | 0.1128 |  | -0.069 | 0.93 | 0.79-1.10 | 0.3975 |
|  | > 35 | 0.084 | 1.30 | 1.10-1.53 | 0.0021 |  | 0.321 | 1.38 | 1.15-1.65 | 0.0005 |
| Abstinence period (days) | <4 | Reference |  |  |  |  | Reference |  |  |  |
|  | 4-7 | 0.304 | 1.36 | 1.17-1.57 | <.0001 |  | 0.420 | 1.52 | 1.29-1.79 | <.0001 |
|  | >7 | 0.569 | 1.77 | 1.43-2.18 | <.0001 |  | 0.717 | 2.05 | 1.63-2.57 | <.0001 |
| Smoking status (cigarettes /day) | 0 | Reference |  |  |  |  | Reference |  |  |  |
|  | <10 | -2.60 | 0.07 | 0.05-0.12 | <.0001 |  | -2.622 | 0.07 | 0.05-0.11 | <.0001 |
|  | 10-20 | 0.25 | 1.28 | 1.05-1.56 | 0.0138 |  | 0.306 | 1.36 | 1.10-1.67 | 0.0041 |
|  | >20 | 2.29 | 9.91 | 7.03-13.97 | <.0001 |  | 2.412 | 11.16 | 7.82-15.93 | <.0001 |
| Alcohol consumption (g/day) | 0 | Reference |  |  |  |  | Reference |  |  |  |
|  | < 9.9 | -0.256 | 0.77 | 0.68-0.88 | <.0001 |  | -0.252 | 0.78 | 0.67-0.90 | 0.0006 |
|  | 10-18.9 | -0.298 | 0.74 | 0.53-1.04 | 0.0793 |  | -0.235 | 0.79 | 0.53-1.18 | 0.2462 |
|  | >19 | 0.206 | 1.23 | 0.11-13.57 | 0.8663 |  | -0.553 | 0.58 | 0.03-10.05 | 0.7049 |
| Staying_up_late | never | Reference |  |  |  |  |  |  |  |  |
|  | Occasionally | -0.039 | 0.96 | 0.82-1.12 | 0.6258 |  |  |  |  |  |
|  | Often | -0.162 | 0.85 | 0.71-1.02 | 0.0782 |  |  |  |  |  |
|  | Always | -0.129 | 0.88 | 0.70-1.11 | 0.2847 |  |  |  |  |  |
| Sleeplessness | never | Reference |  |  |  |  |  |  |  |  |
|  | Occasionally | 0.088 | 1.10 | 0.95-1.25 | 0.2060 |  |  |  |  |  |
|  | Often | 0.216 | 1.24 | 0.99-1.55 | 0.0588 |  |  |  |  |  |
|  | Always | 0.244 | 1.28 | 0.73-2.23 | 0.3940 |  |  |  |  |  |
| Consumption of pungent food | never | Reference |  |  |  |  |  |  |  |  |
|  | Occasionally | -0.239 | 0.79 | 0.67-0.93 | 0.0056 |  |  |  |  |  |
|  | Often | -0.324 | 0.72 | 0.60-0.88 | 0.0011 |  |  |  |  |  |
|  | Always | -0.316 | 0.73 | 0.52-1.03 | 0.0738 |  |  |  |  |  |
| Intensity of sports activity (times/week) | 0 | Reference |  |  |  |  |  |  |  |  |
|  | <1 | -0.170 | 0.84 | 0.68-1.04 | 0.1139 |  |  |  |  |  |
|  | 2-3 | -0.154 | 0.86 | 0.69-1.06 | 0.1541 |  |  |  |  |  |
|  | 4-5 | -0.199 | 0.82 | 0.59-1.13 | 0.2267 |  |  |  |  |  |
|  | >5 | 0.033 | 1.03 | 0.59-1.83 | 0.9095 |  |  |  |  |  |
| Sedentary lifestyle | No | Reference |  |  |  |  |  |  |  |  |
|  | Yes | -0.124 | 0.88 | 0.77-1.01 | 0.0715 |  |  |  |  |  |
| Work in hot conditions | No | Reference |  |  |  |  | Reference |  |  |  |
|  | Yes | 0.385 | 1.47 | 1.13-1.91 | 0.0042 |  | 0.404 | 1.50 | 1.11-2.03 | 0.0089 |
| Sauna use in the last 3 months | No | Reference |  |  |  |  |  |  |  |  |
|  | Yes | -0.005 | 1.00 | 0.63-1.55 | 0.9834 |  |  |  |  |  |
| Exposure to radioactivity (Source) | None | Reference |  |  |  |  |  |  |  |  |
|  | Computer | -0.190 | 0.83 | 0.72-0.95 | 0.0072 |  | -0.229 | 0.80 | 0.68-0.93 | 0.0034 |
|  | Radio | -1.315 | 0.27 | 0.03-2.13 | 0.2128 |  | -1.454 | 0.23 | 0.03-2.19 | 0.2025 |
|  | Others | 0.322 | 1.38 | 0.67-2.83 | 0.3801 |  | 0.332 | 1.39 | 0.64-3.04 | 0.4039 |
