# Supplementary material for: Preliminary prediction of semen quality based on modifiable lifestyle factors by using the XGBoost algorithm
Source: Front Med (Lausanne). 2022 Sep 13;9:811890. doi: 10.3389/fmed.2022.811890 (PMC9514383; doi:10.3389/fmed.2022.811890)
Supplement: Supplementary file 7 [file Table_7.docx]

**Supplementary Table 7.** Univariate and multivariate analyses of total sperm motility

| Variable | Controls |  | Univariate analysis | | |  |  | Multivariate analysis | | |  |
| --- | --- | --- | --- | --- | --- | --- | --- | --- | --- | --- | --- |
|  |  | β | OR | 95%CI | *p-*value |  | β | OR | 95%CI | *p-*value |  |
| Season of semen examination | Spring | Reference |  |  |  |  |  |  |  |  |  |
|  | Summer | 0.213 | 1.24 | 1.04-1.47 | 0.0163 |  | 0.186 | 1.21 | 1.00-1.45 | 0.0503 |  |
|  | Autumn | -0.141 | 0.87 | 0.71-1.06 | 0.1671 |  | -0.148 | 0.86 | 0. 70-1.07 | 0.1747 |  |
|  | Winter | 0.143 | 1.15 | 0.98-1.36 | 0.0931 |  | 0.134 | 1.14 | 0.96-1.37 | 0.1412 |  |
| Age (years) | < 30 | Reference |  |  |  |  | Reference |  |  |  |  |
|  | 30-35 | -0.139 | 0.87 | 0.75-1.01 | 0.0733 |  | -0.092 | 0.91 | 0.77-1.08 | 0.2753 |  |
|  | > 35 | 0.239 | 1.27 | 1.07-1.50 | 0.0055 |  | 0.3017 | 1.35 | 1.13-1.63 | 0.0013 |  |
| Abstinence period (days) | <4 | Reference |  |  |  |  |  |  |  |  |  |
|  | 4-7 | 0.225 | 1.25 | 1.08-1.46 | 0.0038 |  | 0.3164 | 1.37 | 1.16-1.62 | 0.0002 |  |
|  | >7 | 0.423 | 1.53 | 1.23-1.89 | 0.0001 |  | 0.5444 | 1.72 | 1.37-2.17 | <.0001 |  |
| Smoking status (cigarettes /day) | 0 | Reference |  |  |  |  | Reference |  |  |  |  |
|  | <10 | -2.854 | 0.06 | 0.03-0.10 | <.0001 |  | -2.8597 | 0.06 | 0.03 -0.10 | <.0001 |  |
|  | 10-20 | 0.1036 | 1.109 | 0.90-1.36 | 0.3232 |  | 0.1580 | 1.17 | 0.94-1.46 | 0.1560 |  |
|  | >20 | 2.22 | 9.19 | 6.61-12.78 | <.0001 |  | 2.3366 | 10.35 | 7.35-14.56 | <.0001 |  |
| Alcohol consumption (g/day) | 0 | Reference |  |  |  |  |  |  |  |  |  |
|  | < 9.9 | -0.294 | 0.75 | 0.65-0.85 | <.0001 |  | -0.2402 | 0.79 | 0.68-0.92 | 0.0018 |  |
|  | 10-18.9 | -0.380 | 0.68 | 0.48-0.97 | 0.0333 |  | -0.2839 | 0.75 | 0.49-1.15 | 0.1865 |  |
|  | >19 | 0.296 | 1.35 | 0.12-14.85 | 0.8091 |  | -0.4131 | 0.66 | 0.04-11.91 | 0.7794 |  |
| Staying_up_late | never | Reference |  |  |  |  |  |  |  |  |  |
|  | Occasionally | -0.064 | 0.94 | 0.80-1.10 | 0.4291 |  |  |  |  |  |  |
|  | Often | -0.236 | 0.79 | 0.66-0.95 | 0.0125 |  |  |  |  |  |  |
|  | Always | -0.172 | 0.84 | 0.66-1.07 | 0.1632 |  |  |  |  |  |  |
| Sleeplessness | never | Reference |  |  |  |  |  |  |  |  |  |
|  | Occasionally | 0.0653 | 1.07 | 0.93-1.23 | 0.3598 |  |  |  |  |  |  |
|  | Often | 0.1189 | 1.13 | 0.89-1.42 | 0.3172 |  |  |  |  |  |  |
|  | Always | 0.2538 | 1.29 | 0.73-2.28 | 0.3825 |  |  |  |  |  |  |
| Consumption of pungent food | never | Reference |  |  |  |  | Reference |  |  |  |  |
|  | Occasionally | -0.324 | 0.72 | 0.61-0.86 | 0.0002 |  | -0.271 | 0.76 | 0.64-0.92 | 0.0038 |  |
|  | Often | -0.425 | 0.65 | 0.54-0.80 | <.0001 |  | -0.290 | 0.75 | 0.60-0.94 | 0.0109 |  |
|  | Always | -0.414 | 0.66 | 0.46 -0.94 | 0.0228 |  | -0.034 | 0.97 | 0.64-1.45 | 0.8711 |  |
| Intensity of sports activity (times/week) | 0 | Reference |  |  |  |  |  |  |  |  |  |
|  | <1 | -0.15 | 0.86 | 0.69-1.07 | 0.1717 |  |  |  |  |  |  |
|  | 2-3 | -0.120 | 0.89 | 0.71-1.10 | 0.2797 |  |  |  |  |  |  |
|  | 4-5 | -0.103 | 0.90 | 0.65-1.25 | 0.5367 |  |  |  |  |  |  |
|  | >5 | -0.159 | 0.85 | 0.46-1.57 | 0.6108 |  |  |  |  |  |  |
| Sedentary lifestyle | No | Reference |  |  |  |  |  |  |  |  |  |
|  | Yes | -0.137 | 0.87 | 0.76-1.00 | 0.0528 |  |  |  |  |  |  |
| Work in hot conditions | No |  |  |  |  |  | Reference |  |  |  |  |
|  | Yes | 0.445 | 1.56 | 1.20-2.04 | 0.0011 |  | 0.4863 | 1.63 | 1.20 -2.21 | 0.0018 |  |
| Sauna use in the last 3 months | No | Reference |  |  |  |  |  |  |  |  |  |
|  | Yes | -0.055 | 0.95 | 0.06-1.50 | 0.8158 |  |  |  |  |  |  |
| Exposure to radioactivity (Source) | None | Reference |  |  |  |  |  |  |  |  |  |
|  | Computer | -0.197 | 0.82 | 0.71-0.95 | 0.0063 |  | -0.2209 | 0.80 | 0.69-0.94 | 0.0059 |  |
|  | Radio | -1.214 | 0.30 | 0.04-2.35 | 0.2503 |  | -1.3443 | 0.26 | 0.03-2.38 | 0.2339 |  |
|  | Others | 0.2900 | 1.34 | 0.64-2.78 | 0.4384 |  | 0.3510 | 1.42 | 1.42-0.64 | 0.3852 |  |
