# Supplementary material for: Preliminary prediction of semen quality based on modifiable lifestyle factors by using the XGBoost algorithm
Source: Front Med (Lausanne). 2022 Sep 13;9:811890. doi: 10.3389/fmed.2022.811890 (PMC9514383; doi:10.3389/fmed.2022.811890)
Supplement: Supplementary file 8 [file Table_8.docx]

**Supplementary Table 8.** Univariate and multivariate analyses of sperm morphology

| Variable | Controls |  | Univariate analysis | | |  |  | Multivariate analysis | | |
| --- | --- | --- | --- | --- | --- | --- | --- | --- | --- | --- |
|  |  | β | OR | 95%CI | *p-*value |  | β | OR | 95%CI | *p-*value |
| Season of semen examination | Spring | Reference |  |  |  |  |  |  |  |  |
|  | Summer | 0.014 | 1.01 | 0.78-1.31 | 0.9178 |  |  |  |  |  |
|  | Autumn | -0.095 | 0.91 | 0.68-1.22 | 0.5222 |  |  |  |  |  |
|  | Winter | -0.115 | 0.89 | 0.67-1.19 | 0.4371 |  |  |  |  |  |
| Age (years) | < 30 | Reference |  |  |  |  |  |  |  |  |
|  | 30-35 | 0.019 | 1.02 | 0.79-1.31 | 0.8841 |  |  |  |  |  |
|  | > 35 | 0.266 | 1.31 | 0.99-1.72 | 0.0578 |  |  |  |  |  |
| Abstinence period (days) | <4 | Reference |  |  |  |  |  |  |  |  |
|  | 4-7 | 0.093 | 0.91 | 0.73-1.15 | 0.4269 |  |  |  |  |  |
|  | >7 | -0.148 | 0.86 | 0.61-1.22 | 0.4055 |  |  |  |  |  |
| Smoking status (cigarettes /day) | 0 | Reference |  |  |  |  |  |  |  |  |
|  | <10 | -0.637 | 0.53 | 0.39-0.73 | <.0001 |  |  |  |  |  |
|  | 10-20 | 0.29 | 1.34 | 0.99-1.81 | 0.0613 |  |  |  |  |  |
|  | >20 | 1.099 | 3 | 1.761-5.118 | <.0001 |  |  |  |  |  |
| Alcohol consumption (g/day) | 0 | Reference |  |  |  |  |  |  |  |  |
|  | < 9.9 | -0.162 | 0.85 | 0.69-1.05 | 0.1278 |  |  |  |  |  |
|  | 10-18.9 | -0.266 | 0.77 | 0.45-1.31 | 0.3305 |  |  |  |  |  |
|  | >19 | 0.325 | 1.38 | 0.14-13.35 | 0.7789 |  |  |  |  |  |
| Staying_up_late | never | Reference |  |  |  |  |  |  |  |  |
|  | Occasionally | -0.032 | 0.97 | 0.75-1.25 | 0.8093 |  |  |  |  |  |
|  | Often | 0.043 | 1.04 | 0.78-1.39 | 0.7701 |  |  |  |  |  |
|  | Always | 0.132 | 1.14 | 0.78-1.67 | 0.4952 |  |  |  |  |  |
| Sleeplessness | never | Reference |  |  |  |  |  |  |  |  |
|  | Occasionally | -0.032 | 0.97 | 0.78-1.21 | 0.7754 |  |  |  |  |  |
|  | Often | -0.191 | 0.83 | 0.56-1.22 | 0.3356 |  |  |  |  |  |
|  | Always | 0.291 | 1.34 | 0.57-3.15 | 0.5047 |  |  |  |  |  |
| Consumption of pungent food | never | Reference |  |  |  |  |  |  |  |  |
|  | Occasionally | 0.033 | 1.03 | 0.77-1.38 | 0.8271 |  |  |  |  |  |
|  | Often | 0.145 | 1.16 | 0.83-1.60 | 0.3837 |  |  |  |  |  |
|  | Always | -0.149 | 0.86 | 0.48-1.54 | 0.6138 |  |  |  |  |  |
| Intensity of sports activity (times/week) | 0 | Reference |  |  |  |  |  |  |  |  |
|  | <1 | -0.062 | 0.94 | 0.66-1.33 | 0.7278 |  |  |  |  |  |
|  | 2-3 | -0.172 | 0.84 | 0.59-1.20 | 0.3360 |  |  |  |  |  |
|  | 4-5 | 0.088 | 1.09 | 0.67-1.79 | 0.7282 |  |  |  |  |  |
|  | >5 | -0.07 | 0.93 | 0.34-2.59 | 0.8928 |  |  |  |  |  |
| Sedentary lifestyle | No | Reference |  |  |  |  |  |  |  |  |
|  | Yes | 0.005 | 1.01 | 0.80-1.26 | 0.9633 |  |  |  |  |  |
| Work in hot conditions | No | Reference |  |  |  |  |  |  |  |  |
|  | Yes | 0.1 | 1.11 | 0.69-1.76 | 0.6756 |  |  |  |  |  |
| Sauna use in the last 3 months | No | Reference |  |  |  |  |  |  |  |  |
|  | Yes | 0.038 | 1.04 | 0.50-2.16 | 0.9196 |  |  |  |  |  |
| Exposure to radioactivity (Source) | None | Reference |  |  |  |  |  |  |  |  |
|  | Computer | -0.189 | 0.83 | 0.66-1.04 | 0.0978 |  |  |  |  |  |
|  | Radio | 1.084 | 2.96 | 0.65-13.36 | 0.1593 |  |  |  |  |  |
|  | Others | -0.169 | 0.85 | 0.24-2.98 | 0.7928 |  |  |  |  |  |
