# Supplementary material for: Preliminary prediction of semen quality based on modifiable lifestyle factors by using the XGBoost algorithm
Source: Front Med (Lausanne). 2022 Sep 13;9:811890. doi: 10.3389/fmed.2022.811890 (PMC9514383; doi:10.3389/fmed.2022.811890)
Supplement: Supplementary file 9 [file Table_9.docx]

**Supplementary Table 9.** Univariate and multivariate analyses of DFI

| Variable | Controls |  | Univariate analysis | | |  |  | Multivariate analysis | | |
| --- | --- | --- | --- | --- | --- | --- | --- | --- | --- | --- |
|  |  | β | OR | 95%CI | *p-*value |  | β | OR | 95%CI | *p-*value |
| Season of semen examination | Spring | Reference |  |  |  |  |  |  |  |  |
|  | Summer | 0.179 | 1.2 | 0.83-1.73 | 0.3409 |  |  |  |  |  |
|  | Autumn | 0.135 | 1.15 | 0.78-1.68 | 0.4901 |  |  |  |  |  |
|  | Winter | 0.065 | 1.07 | 0.74-1.54 | 0.7273 |  |  |  |  |  |
| Age (years) | < 30 | Reference |  |  |  |  | Reference |  |  |  |
|  | 30-35 | 0.904 | 2.47 | 1.54-53.95 | 0.0002 |  | 0.893 | 2.44 | 1.52-3.93 | 0.0002 |
|  | > 35 | 1.788 | 5.98 | 3.76-9.51 | <.0001 |  | 1.699 | 5.47 | 3.41- 8.76 | <.0001 |
| Abstinence period (days) | <4 | Reference |  |  |  |  |  |  |  |  |
|  | 4-7 | 10.1678 | >999.999 | <0.001->999.999 | 0.9705 |  | 0.628 | 1.87 | 1.30-2.71 | 0.0008 |
|  | >7 | 10.7604 | >999.999 | <0.001->999.999 | 0.9688 |  | 1.2845 | 3.61 | 2.27- 5.75 | <.0001 |
| Smoking status (cigarettes /day) | 0 | Reference |  |  |  |  | Reference |  |  |  |
|  | <10 | -0.869 | 0.42 | 0.27-0.64 | <.0001 |  | -0.86 | 0.42 | 0.27- 0.66 | 0.0002 |
|  | 10-20 | -0.094 | 0.91 | 0.58-1.44 | 0.6866 |  | -0.002 | 1.00 | 0.61- 1.62 | 0.9946 |
|  | >20 | 0.788 | 2.2 | 1.04-4.65 | 0.0393 |  | 0.756 | 2.121 | 0.95- 4.73 | 0.0659 |
| Alcohol consumption (g/day) | 0 | Reference |  |  |  |  |  |  |  |  |
|  | < 9.9 | -0.47 | 0.63 | 0.47-0.83 | 0.0012 |  |  |  |  |  |
|  | 10-18.9 | -0.376 | 0.69 | 0.35-1.36 | 0.2807 |  |  |  |  |  |
|  | >19 | -11.441 | <0.001 | <0.001，>999.999 | 0.9770 |  |  |  |  |  |
| Staying_up_late | never | Reference |  |  |  |  |  |  |  |  |
|  | Occasionally | -0.153 | 0.86 | 0.63-1.18 | 0.3432 |  | -0.039 | 0.961 | 0.69-1.34 | 0.8153 |
|  | Often | -0.251 | 0.78 | 0.53-1.13 | 0.1878 |  | -0.026 | 0.974 | 0.65-1.45 | 0.8986 |
|  | Always | -1.367 | 0.26 | 0.12-0.54 | 0.0004 |  | -1.217 | 0.296 | 0.14-0.64 | 0.0020 |
| Sleeplessness | never | Reference |  |  |  |  |  |  |  |  |
|  | Occasionally | 0.195 | 1.22 | 0.91-1.62 | 0.1854 |  |  |  |  |  |
|  | Often | 0.115 | 0.89 | 0.53-1.50 | 0.6656 |  |  |  |  |  |
|  | Always | 0.557 | 1.75 | 0.64-4.75 | 0.2754 |  |  |  |  |  |
| Consumption of pungent food | never | Reference |  |  |  |  |  |  |  |  |
|  | Occasionally | -0.14 | 0.87 | 0.61-1.23 | 0.4343 |  |  |  |  |  |
|  | Often | 0.592 | 0.55 | 0.36-0.86 | 0.0080 |  |  |  |  |  |
|  | Always | -0.284 | 0.75 | 0.35-1.62 | 0.4665 |  |  |  |  |  |
| Intensity of sports activity (times/week) | 0 | Reference |  |  |  |  |  |  |  |  |
|  | <1 | 0.116 | 1.12 | 0.67-1.88 | 0.6581 |  |  |  |  |  |
|  | 2-3 | 0.309 | 1.36 | 0.82-2.26 | 0.2311 |  |  |  |  |  |
|  | 4-5 | 0.734 | 2.08 | 1.10-3.93 | 0.0236 |  |  |  |  |  |
|  | >5 | -0.883 | 0.41 | 0.05-3.26 | 0.4017 |  |  |  |  |  |
| Sedentary lifestyle | No | Reference |  |  |  |  |  |  |  |  |
|  | Yes | 0.233 | 1.26 | 0.93-1.72 | 0.1374 |  |  |  |  |  |
| Work in hot conditions | No | Reference |  |  |  |  |  |  |  |  |
|  | Yes | -0.093 | 0.91 | 0.46-1.79 | 0.7875 |  |  |  |  |  |
| Sauna use in the last 3 months | No | Reference |  |  |  |  |  |  |  |  |
|  | Yes | 0.190 | 1.21 | 0.50-2.93 | 0.6744 |  |  |  |  |  |
| Exposure to radioactivity (Source) | None | Reference |  |  |  |  |  |  |  |  |
|  | Computer | 0.281 | 1.32 | 0.97-1.81 | 0.0798 |  |  |  |  |  |
|  | Radio | 12.033 | <0.001 | <0.001，>999.999 | 0.979 |  |  |  |  |  |
|  | Others | 0.424 | 1.53 | 0.33-7.14 | 0.5904 |  |  |  |  |  |

| Items | Fill the following blanks or select the options | | | | | | | | The criterion of Categorical variable split | | | | | |
| --- | --- | --- | --- | --- | --- | --- | --- | --- | --- | --- | --- | --- | --- | --- |
| Date (Day/Month/Year) |  | | | | | | | | Spring (March 21st-Jun 20st) | Summer (June 21st-Septemnber 22st) | Autumn (September 23st-December 21st) | Winter (December 22st-March 20st) | | |
| Age (years) |  | | | | | | | | < 30 | 30-35 | > 35 | | | |
| Abstinence period (day) |  | | | | | | | | < 4 | 4-7 | > 7 | | | |
| Smoking in the latest two years (cigarettes /day) |  | | | | | | | | 0 | <9 | 10-20 | | >20 | |
| Alcohol consumption per day in the latest two years | Light beer (ethanol:<4% vol, 330ml/botton or can) | | Regular beer (ethanol:≥4% vol,330ml/botton or can) | Wine (4 ounce glass) | | Spirits (ethanol:<30% vol,10ml/glass) | Spirits (ethanol:30-50% vol,10ml/glass) | Liquor (ethanol:>50% vol, 10ml/glass) | 0 | < 9.9g/day | 9.9-19 g/day | | >19 g/day | |
|  |  |  | |  | |  |  |  |  |  |  |  |  |  |
| Bedtimes (PM) |  | | | | | | | | < 10 PM | 10-10:59 PM | 11-12 PM | | >12PM | |
| Sleeplessness (score of ISI) ^&^ | Please fill the scale of Insomnia Severity Index (ISI) in Supplementary table XXX. | | | | | | | | 0-7 | 8-14 | 15-21 | | 22-28 | |
| Pungent-flavor food consumption | 🞏Never; 🞏<1 day per week; 🞏1-5 days a week; 🞏> 5 days a week | | | | | | | | Never | <1 day per week (occasionally) | 1-5 days a week (often) | | > 5 days a week (aways) | |
| Sports (TEEQ-C)# | Please fill the Total Energy Expenditure Questionnaire (TEEQ-C) in Supplementary table XXX. | | | | | | | | ≤1.99METs | 2-2.99METs | 3-3.99METs | | 4-5.99METs | ≥6 METs |
| Sedentary (h/day) |  | | | | | | | | < 5 | ≥5 | | | | |
| Does your work under high environment temperature exposure (WBGT≥ 25℃)? | 🞏 No | | | | Yes | | | | No | Yes | | | | |
|  |  |  |  |  | I’m a (🞏 outdoor construction worker; 🞏steelworker; 🞏truck driver; 🞏others____), and I have received the high-temperature allowance in the last three months. | | | |  |  |  |  |  |  |
| Sauna usage in the latest 3 months | 🞏 No | | | | 🞏 Yes | | | | No | Yes | | | | |
| Do you often under electromagnetic radiation exposure? | 🞏 NO | | | | Yes | | | | No | Computer | Radio | | Others | |
|  |  |  |  |  | 🞏computer; 🞏Radio; 🞏others___ | | | |  |  |  |  |  |  |

# The intensity categories were classified by the criterion of Chu’s article (PMID: 26327457). & The intensity categories were classified by the criterion of Hu’s article (PMID: 25957853)
