# Supplementary material for: Preliminary prediction of semen quality based on modifiable lifestyle factors by using the XGBoost algorithm
Source: Front Med (Lausanne). 2022 Sep 13;9:811890. doi: 10.3389/fmed.2022.811890 (PMC9514383; doi:10.3389/fmed.2022.811890)
Supplement: Supplementary file 10 [file Table_10.docx]

**Supplementary Table 10.** Correlations among general information parameters

| **Correlations** | | | | | | | | | | | | | | | |
| --- | --- | --- | --- | --- | --- | --- | --- | --- | --- | --- | --- | --- | --- | --- | --- |
|  | | | Season of semen examination | Age | Abstinance_period | Smoking status | Alcohol consumption | Staying_up_late | Sleeplessness | Consumption of pungent food | Intensity of sports activity | Sedentary lifestyle | Work in hot conditions | Sauna use in the last 3 months | Exposure to radioactivity |
|  | Season of semen examination | Correlation Coefficient | 1.000 | .006 | .009 | -.026 | -.020 | -.005 | -.009 | -.018 | -.013 | .027 | -.051^**^ | .025 | .020 |
|  |  | Sig. (2-tailed) |  | .674 | .509 | .061 | .144 | .714 | .541 | .195 | .336 | .051 | .000 | .076 | .154 |
|  | Age | Correlation Coefficient | .006 | 1.000 | .066^**^ | -.027 | .049^**^ | -.074^**^ | .045^**^ | -.060^**^ | .052^**^ | .043^**^ | -.070^**^ | .011 | .012 |
|  |  | Sig. (2-tailed) | .674 |  | .000 | .054 | .000 | .000 | .001 | .000 | .000 | .002 | .000 | .416 | .378 |
|  | Abstinance_period | Correlation Coefficient | .009 | .066^**^ | 1.000 | -.038^**^ | -.029^*^ | -.055^**^ | -.012 | -.041^**^ | .014 | -.022 | .022 | -.006 | -.020 |
|  |  | Sig. (2-tailed) | .509 | .000 |  | .006 | .035 | .000 | .378 | .004 | .312 | .111 | .121 | .686 | .154 |
|  | Smoking status | Correlation Coefficient | -.026 | -.027 | -.038^**^ | 1.000 | .316^**^ | .185^**^ | .132^**^ | .184^**^ | -.022 | -.088^**^ | .073^**^ | .062^**^ | -.142^**^ |
|  |  | Sig. (2-tailed) | .061 | .054 | .006 |  | .000 | .000 | .000 | .000 | .113 | .000 | .000 | .000 | .000 |
|  | Alcohol consumption | Correlation Coefficient | -.020 | .049^**^ | -.029^*^ | .316^**^ | 1.000 | .238^**^ | .160^**^ | .287^**^ | .007 | -.017 | .041^**^ | .045^**^ | -.028^*^ |
|  |  | Sig. (2-tailed) | .144 | .000 | .035 | .000 |  | .000 | .000 | .000 | .594 | .227 | .004 | .001 | .047 |
|  | Staying_up_late | Correlation Coefficient | -.005 | -.074^**^ | -.055^**^ | .185^**^ | .238^**^ | 1.000 | .310^**^ | .342^**^ | -.067^**^ | .078^**^ | .087^**^ | .067^**^ | .034^*^ |
|  |  | Sig. (2-tailed) | .714 | .000 | .000 | .000 | .000 |  | .000 | .000 | .000 | .000 | .000 | .000 | .015 |
|  | Sleeplessnss | Correlation Coefficient | -.009 | .045^**^ | -.012 | .132^**^ | .160^**^ | .310^**^ | 1.000 | .233^**^ | .009 | .056^**^ | .055^**^ | .046^**^ | .003 |
|  |  | Sig. (2-tailed) | .541 | .001 | .378 | .000 | .000 | .000 |  | .000 | .529 | .000 | .000 | .001 | .807 |
|  | Consumption of pungent food | Correlation Coefficient | -.018 | -.060^**^ | -.041^**^ | .184^**^ | .287^**^ | .342^**^ | .233^**^ | 1.000 | -.048^**^ | .061^**^ | .081^**^ | .067^**^ | .046^**^ |
|  |  | Sig. (2-tailed) | .195 | .000 | .004 | .000 | .000 | .000 | .000 |  | .001 | .000 | .000 | .000 | .001 |
|  | Intensity of sports activity | Correlation Coefficient | -.013 | .052^**^ | .014 | -.022 | .007 | -.067^**^ | .009 | -.048^**^ | 1.000 | -.119^**^ | -.005 | .020 | -.016 |
|  |  | Sig. (2-tailed) | .336 | .000 | .312 | .113 | .594 | .000 | .529 | .001 |  | .000 | .699 | .162 | .247 |
|  | Sedentary lifestyle | Correlation Coefficient | .027 | .043^**^ | -.022 | -.088^**^ | -.017 | .078^**^ | .056^**^ | .061^**^ | -.119^**^ | 1.000 | -.134^**^ | .008 | .438^**^ |
|  |  | Sig. (2-tailed) | .051 | .002 | .111 | .000 | .227 | .000 | .000 | .000 | .000 |  | .000 | .563 | .000 |
|  | Work in hot conditions | Correlation Coefficient | -.051^**^ | -.070^**^ | .022 | .073^**^ | .041^**^ | .087^**^ | .055^**^ | .081^**^ | -.005 | -.134^**^ | 1.000 | .082^**^ | -.109^**^ |
|  |  | Sig. (2-tailed) | .000 | .000 | .121 | .000 | .004 | .000 | .000 | .000 | .699 | .000 |  | .000 | .000 |
|  | Sauna use in the last 3 months | Correlation Coefficient | .025 | .011 | -.006 | .062^**^ | .045^**^ | .067^**^ | .046^**^ | .067^**^ | .020 | .008 | .082^**^ | 1.000 | .000 |
|  |  | Sig. (2-tailed) | .076 | .416 | .686 | .000 | .001 | .000 | .001 | .000 | .162 | .563 | .000 |  | .997 |
|  | Exposure to radioactivity | Correlation Coefficient | .020 | .012 | -.020 | -.142^**^ | -.028^*^ | .034^*^ | .003 | .046^**^ | -.016 | .438^**^ | -.109^**^ | .000 | 1.000 |
|  |  | Sig. (2-tailed) | .154 | .378 | .154 | .000 | .047 | .015 | .807 | .001 | .247 | .000 | .000 | .997 |  |
| **. Correlation is significant at the 0.01 level (2-tailed). | | | | | | | | | | | | | | | |
| *. Correlation is significant at the 0.05 level (2-tailed). | | | | | | | | | | | | | | | |
